# WNT signaling in the tumor microenvironment promotes immunosuppression in murine pancreatic cancer

**DOI:** 10.1084/jem.20220503

**Published:** 2022-10-14

**Authors:** Wenting Du, Rosa E. Menjivar, Katelyn L. Donahue, Padma Kadiyala, Ashley Velez-Delgado, Kristee L. Brown, Hannah R. Watkoske, Xi He, Eileen S. Carpenter, Christina V. Angeles, Yaqing Zhang, Marina Pasca di Magliano

**Affiliations:** 1 Department of Surgery, University of Michigan, Ann Arbor, MI; 2 Cellular and Molecular Biology Program, University of Michigan, Ann Arbor, MI; 3 Cancer Biology Program, University of Michigan, Ann Arbor, MI; 4 Immunology Program, University of Michigan, Ann Arbor, MI; 5 Department of Cell and Developmental Biology, University of Michigan, Ann Arbor, MI; 6 College of Literature, Science, and the Arts, University of Michigan, Ann Arbor, MI; 7 Department of Internal Medicine, Division of Gastroenterology, University of Michigan, Ann Arbor, MI; 8 Rogel Cancer Center, University of Michigan, Ann Arbor, MI

## Abstract

Pancreatic ductal adenocarcinoma (PDA) is associated with activation of WNT signaling. Whether this signaling pathway regulates the tumor microenvironment has remained unexplored. Through single-cell RNA sequencing of human pancreatic cancer, we discovered that tumor-infiltrating CD4^+^ T cells express *TCF7*, encoding for the transcription factor TCF1. We conditionally inactivated *Tcf7* in CD4 expressing T cells in a mouse model of pancreatic cancer and observed changes in the tumor immune microenvironment, including more CD8^+^ T cells and fewer regulatory T cells, but also compensatory upregulation of PD-L1. We then used a clinically available inhibitor of Porcupine, a key component of WNT signaling, and observed similar reprogramming of the immune response. WNT signaling inhibition has limited therapeutic window due to toxicity, and PD-L1 blockade has been ineffective in PDA. Here, we show that combination targeting reduces pancreatic cancer growth in an experimental model and might benefit the treatment of pancreatic cancer.

## Teaser

Inactivation of *TCF7,* a WNT transcriptional mediator, in CD4^+^ T cells or pharmacological inhibition of ligand-mediated WNT signaling results in increased CD8^+^ T cell infiltration and reduced pancreatic cancer growth, identifying WNT signaling as a regulator of the pancreatic cancer immune microenvironment.

## Introduction

Pancreatic cancer is a deadly malignancy with a 5-yr survival rate of 11% (Siegel et al., 2022). Notably, even in patients diagnosed with local disease who undergo successful resection, tumor relapse with treatment-resistant disease occurs with a frequency of about 80% (Groot et al., 2018; Jones et al., 2019; Ying et al., 2016). Current chemotherapeutic combinations provided for the treatment of pancreatic cancer include either FOLFIRINOX or Gemcitabine/Abraxane, but not all patients can tolerate their administration, and their efficacy is unfortunately limited (Mizrahi et al., 2020). The advent of immunotherapy has revolutionized treatment for several solid tumors, but it has yet to benefit most pancreatic cancer patients. This may be due to the extensive tumor microenvironment (TME) in pancreatic cancer, which includes abundant immune cells mostly with immune suppressive function (Binnewies et al., 2018; Du et al., 2021; Morrison et al., 2018) and is established during the earliest stages of carcinogenesis (Clark et al., 2007). Therefore, a stronger understanding of the causes of immunosuppression in this disease is urgently needed.

Pancreatic ductal adenocarcinoma (PDA), the most common type of pancreatic cancer, is almost invariably associated with oncogenic mutations in the *KRAS* gene. Further, PDA is associated with inappropriate activation of embryonic signaling pathways that are normally quiescent in the adult pancreas, including Hedgehog, Notch, and WNT (Berman et al., 2003; De La O et al., 2008; Miyamoto et al., 2003; Morris et al., 2010; Pasca di Magliano et al., 2006; Thayer et al., 2003). Genomic studies have also revealed consistent activation of core signaling pathways, including WNT signaling, in pancreatic cancer (Jones et al., 2008). WNT signaling includes canonical and non-canonical pathways; the former is the focus of our work. In the absence of ligands, cytoplasmic β-catenin is targeted for degradation through phosphorylation and ubiquitination (Nusse and Clevers, 2017; Wiese et al., 2018). Canonical WNT signaling is activated by WNT ligands binding to a Frizzled (FZD)/low density lipoprotein receptor-related protein receptor complex, resulting in β-catenin stabilization, cytoplasmic accumulation, and nuclear translocation. In the nucleus, β-catenin binds to transcription factors of the T cell factor (TCF)/lymphoid enhancer factor (LEF) family to form a transcriptional activator complex (Nusse and Clevers, 2017; Wiese et al., 2018). Notably, TCF1 in T cells can act as a transcriptional activator even in the absence of β-catenin due to its ability to bend DNA (Gounari and Khazaie, 2022). Acylation (palmitoylation) of WNT proteins by Porcupine (PORCN), a membrane-bound O-acyltransferase, is essential for WNT ligand secretion and signaling. WNT signaling regulates important crucial biological functions, such as organ development, cell proliferation, differentiation, apoptosis, motility, and survival (Wang et al., 2012). Unlike colorectal cancer, pancreatic cancer is rarely associated with mutations in WNT components. Mutations in the β-catenin gene (*CTNNB1*) are common in mucinous cystic neoplasms, but rare in pancreatic intraepithelial neoplasia (PanIN), the most common precursor lesion of PDA (Ying et al., 2016). Rather, in pancreatic cancer, WNT signaling is more commonly activated through overexpression of WNT ligands (Aguilera and Dawson, 2021) or, in about 5–10% of cases, by mutation of *RNF43,* which sensitizes tumor cells to WNT ligands (Deng et al., 2021; Jiang et al., 2013; Kim et al., 2021; Steinhart et al., 2017).

Canonical WNT signaling has been previously studied in pancreatic cancer (Aguilera and Dawson, 2021). Using a genetic approach, we previously inactivated β-catenin in pancreatic epithelial cells in the context of an oncogenic *Kras*-driven mouse model of pancreatic cancer (*Kras*^*LSL-G12D*^; *Ptf1a-Cre*, commonly referred to as KC) and showed that epithelial WNT signaling is required for PDA initiation and progression (Zhang et al., 2013). Others have demonstrated that epithelial WNT signaling is critical for pancreatic cancer progression, metastasis, and chemoresistance (Jiang et al., 2014; Pai et al., 2016; Ryu et al., 2021; Sano et al., 2016). Intriguingly, activation of WNT signaling has been correlated with non–T cell–inflamed tumor microenvironment and worse prognosis across multiple cancer types (Luke et al., 2019). WNT signaling directly mediates immune evasion in melanoma (Spranger et al., 2015; Spranger and Gajewski, 2015; Spranger and Gajewski, 2018) by hampering dendritic cell–dependent T cell crosspriming (Hong et al., 2016; Murray et al., 1989; Yaguchi et al., 2012). It also regulates the recruitment and function of myeloid-derived suppressor cells (MDSCs), natural killer (NK) cells, and regulatory T cells (Tregs), as well as the expression of several immune checkpoints (Galluzzi et al., 2019). Despite the demonstrated importance of WNT signaling in PDA, its potential role in the TME has remained unexplored.

We recently generated single-cell RNA sequencing (scRNA-seq) data for human pancreatic cancer (Steele et al., 2020). We queried the data to map the expression of WNT signaling components and target genes across the pancreatic cancer microenvironment. Interestingly, we detected expression of WNT components not only in epithelial cells as expected but also in other populations across the microenvironment. In particular, we observed expression of WNT components in tumor-infiltrating lymphocytes (TILs). T cells notably expressed *TCF7,* which encodes for the TCF1 protein, a transcription factor and mediator of WNT signaling that plays an essential role in T cells differentiation (Wang et al., 2018; Zhao et al., 2021). We then queried scRNA-seq datasets from healthy and PDA-bearing mice and similarly detected *Tcf7* in T cells, with an increase in its expression in tumor samples. In the context of cancer, TCF1 has been mainly studied in CD8^+^ T cells, where it is linked to a population of stem-like T cells that provides tumor control (Burger et al., 2021; Han et al., 2021; Hanna et al., 2021; Siddiqui et al., 2019). In melanoma patients, TCF1^+^ CD8^+^ TILs expressed lower levels of PD-1 than TCF1^−^ CD8^+^ TILs (Gattinoni et al., 2011), and the presence of TCF1^+^ CD8^+^ TILs was associated with better clinical response to immune checkpoint blockade therapy (Sade-Feldman et al., 2018). Regarding CD4^+^ T cells, in colon cancer, TCF1 expression in Tregs suppressed tumor growth by inhibiting the transcription of FoxP3-regulated genes (Osman et al., 2021). To functionally dissect the role of T cell *Tcf7* in pancreatic cancer, we generated *Cd4-CreER*^*T2*^;*Tcf7*^*fl/fl*^ mice, where *Tcf7* can be conditionally inactivated in CD4-expressing T cells. Our data show that *Tcf7* inactivation in PDA infiltrating CD4^+^ T cells results in CD8^+^ T cell–driven immune responses. We then evaluated whether pharmacological inhibition of WNT signaling similarly promoted CD8^+^ T cell responses using the PORCN inhibitor LGK974 (blocking all ligand-mediated signaling) and detected similar changes as in the *Tcf7* inactivation model, setting the stage to explore WNT inhibition to sensitize pancreatic cancer to immune checkpoint blockade.

## Results

### *TCF7/Tcf7* is prevalent in CD4^+^ T cells in human and mouse PDA

**Figure S1.**
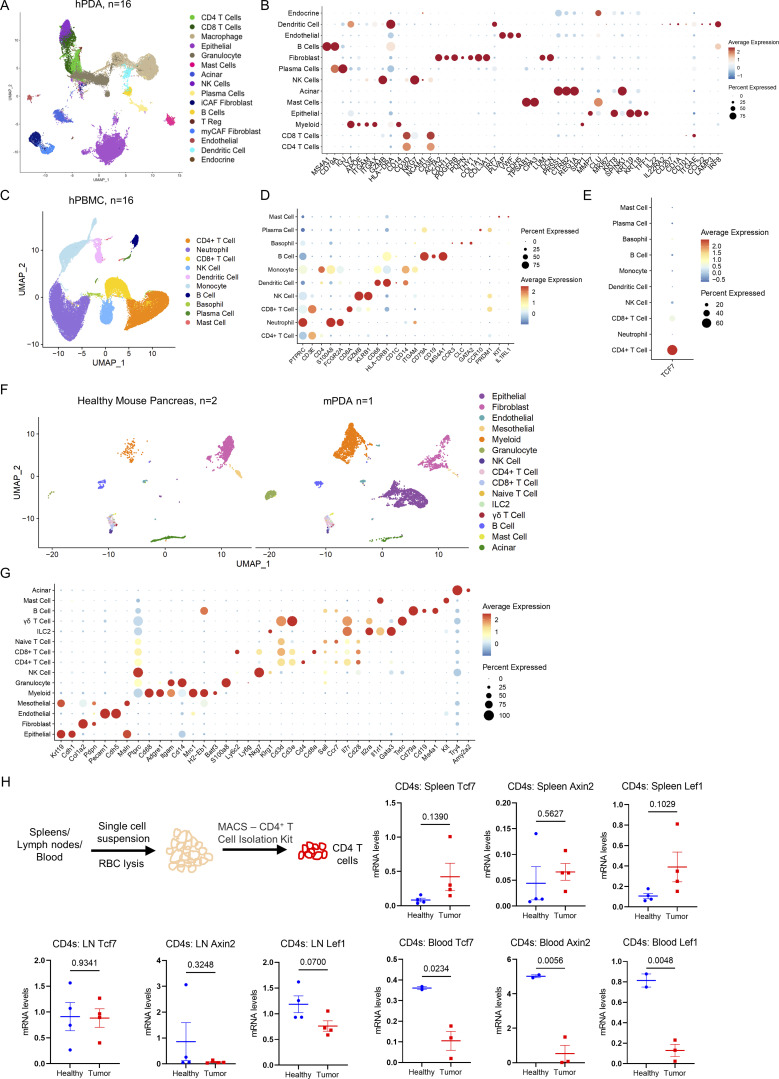
**scRNA-seq data of human and mouse samples. (A)** UMAP representation of identified cell populations in hPDA tumor samples (*n* = 16). **(B)** Dot plot of the key markers used to define the identified cell populations in human samples. **(C)** UMAP representation of identified cell populations in hPDA PBMC. **(D)** Dot plot of the key markers used to define the identified cell populations in hPDA PBMC samples. **(E)** Dot plot of *Tcf7* in all the identified cell populations in hPDA PBMC. **(F)** UMAP representation of identified cell populations in mouse healthy pancreas (*n* = 2) and mPDA (*n* = 1). **(G)** Dot plot of the key markers used to define the identified cell populations in mouse samples. **(H)** qRT-PCR for *Tcf7*, *Lef1*, and *Axin2* expression in CD4^+^ T cells derived from spleen, lymph node (LN), and blood of healthy and tumor-bearing mice (*n* = 2–4/group). Two-tailed Student *t* test (two groups) was performed for comparison between groups. P < 0.05 was considered significant.

**Figure 1.**
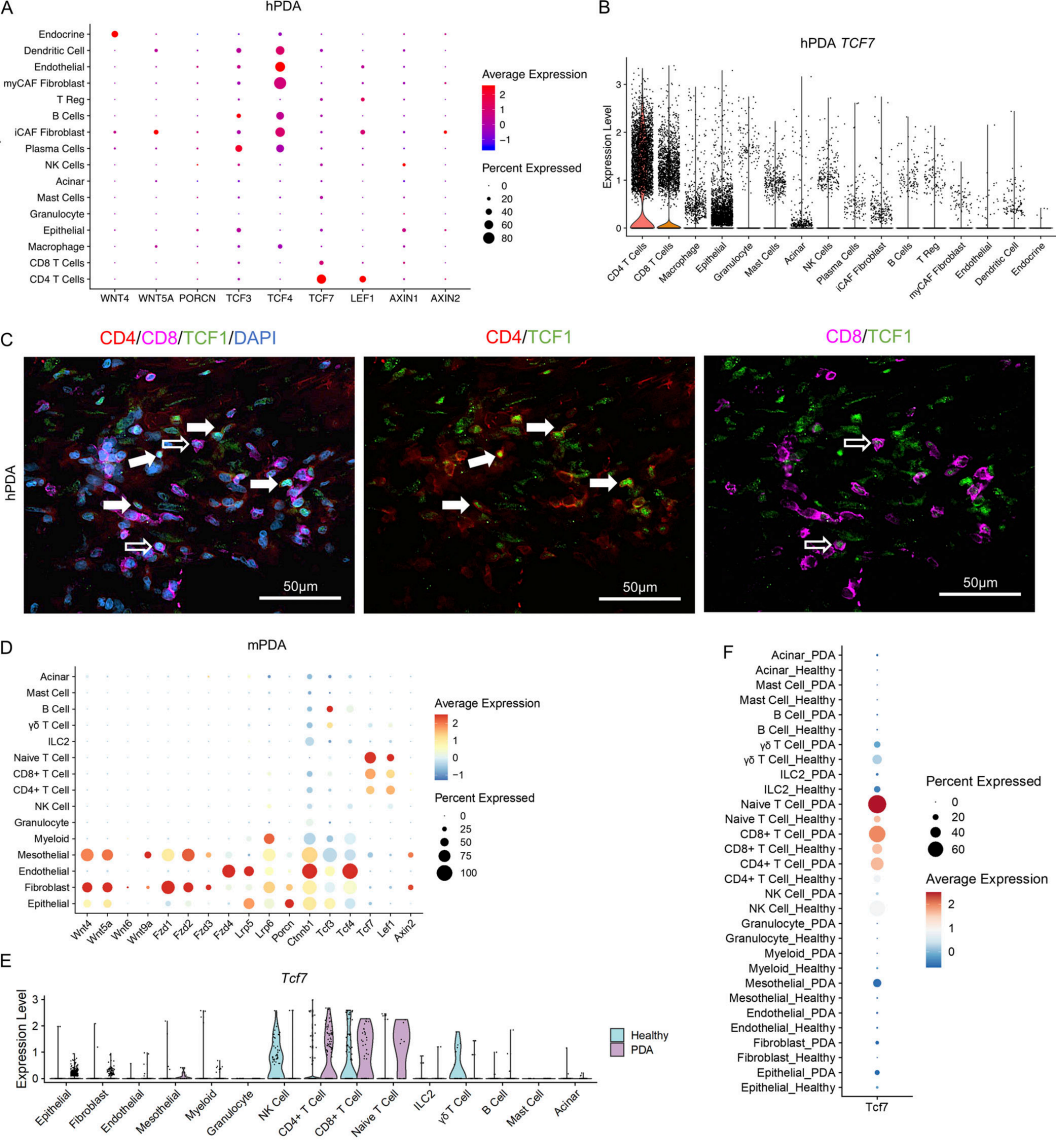
***TCF7/Tcf7* is prevalent in CD4**^**+**^ **T cells in human and mouse PDA. (A)** Dot plot of WNT pathway genes in all identified cell populations in hPDA (*n* = 16). **(B)** Violin plot of *TCF7* in each cell population in hPDA. **(C)** Immunostaining for TCF1 (green), CD4 (red), CD8 (magenta) in human PDA. DAPI nuclear staining is in blue. Full arrows: TCF1 expressing CD4^+^ T cells. Empty arrows: TCF1 expressing CD8^+^ T cells. **(D)** Dot plot of WNT pathway genes in all identified cell populations in mPDA. **(E)** Violin plot of *Tcf7* in all the identified cell populations in healthy mouse pancreas and mPDA. **(F)** Dot plot of *Tcf7* in all the identified cell populations in healthy mouse pancreas and mPDA. iCAF, inflammatory cancer associated fibroblasts; myCAF, myofibroblastic cancer associated fibroblasts; apCAF, antigen-presenting cancer associated fibroblasts.

To map the expression of WNT signaling components across the TME, we queried a scRNA-seq dataset recently generated by our group that includes 16 human PDA (hPDA) samples (Steele et al., 2020). Using uniform manifold approximation and projection (UMAP), we visualized the different cell populations in the samples, including epithelial cells, fibroblasts, myeloid cells, and lymphoid cells (Fig. S1 A). Cell identities for each cluster were determined based on expression of lineage markers (Fig. S1 B), as previously described (Steele et al., 2020). We then plotted the expression of WNT components in each of the major cell populations in the tumors and discovered that the most abundant WNT ligands, namely *WNT4* and *WNT5A*, were highly expressed by fibroblasts and endocrine cells, while WNT/β-catenin endpoint transcription factors *TCF3* and *TCF4* were expressed by epithelial cells, fibroblasts, endothelial cells, myeloid cells, and B cells (Fig. 1 A). The *TCF7* gene, encoding for TCF1, a transcription factor and downstream mediator of WNT signaling, was mostly expressed in CD4^+^ T cells and, to a lesser extent, in CD8^+^ T cells (Fig. 1 A). CD4^+^ T cells also expressed *LEF1*, *AXIN1*, and *AXIN2*, components and target genes of WNT signaling, consistent with pathway activation (Fig. 1 A). We then visualized the same data using a violin plot algorithm to assess differences more finely in *TCF7* expression levels across groups and found that the expression of *TCF7* was both highest and most abundant in CD4^+^ T cells, followed by CD8^+^ T cells (Fig. 1 B). To validate expression of the TCF1 protein, we performed co-immunofluorescent staining on primary human pancreatic cancer samples and found that TCF1 was expressed on CD4^+^ and CD8^+^ T cells (Fig. 1 C). We also investigated the expression of *TCF7* in our human PDA peripheral blood mononuclear cells (PBMCs) dataset (Steele et al., 2020) and found *TCF7* to be abundant in CD4^+^ T cells in comparison to other immune cells (Fig. S1, C–E). Thus, TCF1 is predominantly expressed in T cells in human PDA and could be operating in a WNT-dependent or WNT-independent manner.

To determine whether a similar expression pattern of WNT components was recapitulated in mouse models, we generated scRNA-seq data from healthy pancreata (*n* = 2) and spontaneous tumors from KPC mouse (*Kras*^*LSL-G12D*^*; Trp53*^*LSL-R172H*^*; Ptf1a-Cre*; mPDA, *n* = 1). We visualized cell populations using the UMAP algorithm and identified expected cell populations including epithelial cells, fibroblasts, endothelial cells, mesothelial cells, acinar cells, and immune cells (Fig. S1, F and G). We then mapped the expression of WNT components across different cell populations. As in the human tumors, we found that *Wnt4* and *Wnt5a* were highly expressed by fibroblasts, and to a lesser degree by mesothelial and epithelial cells (Fig. 1 D). *Ctnnb1,* which encodes for β-catenin, was expressed by all cell populations, while *Tcf3* and *Tcf4* were expressed by epithelial cells, fibroblasts, endothelial cells, mesothelial cells, myeloid cells, and B cells (Fig. 1 D). As in the human data, *Tcf7* and *Lef1* were highly expressed by CD4^+^ T cells, CD8^+^ T cells, and naive T cells (Fig. 1 D). Notably, we observed more T cells expressing *Tcf7* and at higher levels in PDA compared with T cells in the healthy pancreas (Fig. 1, E and F). We then isolated CD4^+^ T cells from the spleen, lymph nodes, and blood of healthy mice and tumor-bearing mice and investigated the expression of *Tcf7*, *Lef1*, and *Axin2*. Unlike CD4^+^ T cells infiltrating the tumor, CD4^+^ T cells in secondary lymphoid tissue had either unchanged or lower expression of these WNT components/target genes when compared with tumor-bearing mice within the control group (Fig. S1 H).

### TCF1 signaling in CD4^+^ T cells mediates their immunosuppressive potential

CD4^+^ T cells are a key mediator of immune suppression in pancreatic cancer (Zhang et al., 2014). We and others have previously demonstrated that targeting CD4^+^ T cells prevents PanIN progression due to CD8^+^ T cell–mediated responses (Daley et al., 2016; Jang et al., 2017; McAllister et al., 2014; Ochi et al., 2012). TCF1, originally identified as a T lymphocyte–specific transcription factor, has a critical role in the development and differentiation of T cells (van de Wetering et al., 1991; Wang et al., 2018; Zhao et al., 2021). Given the expression of TCF1 in lymphocytes in human and mouse PDA, we queried whether TCF1 regulates the immunosuppressive function of CD4^+^ T cells in PDA.

**Figure 2.**
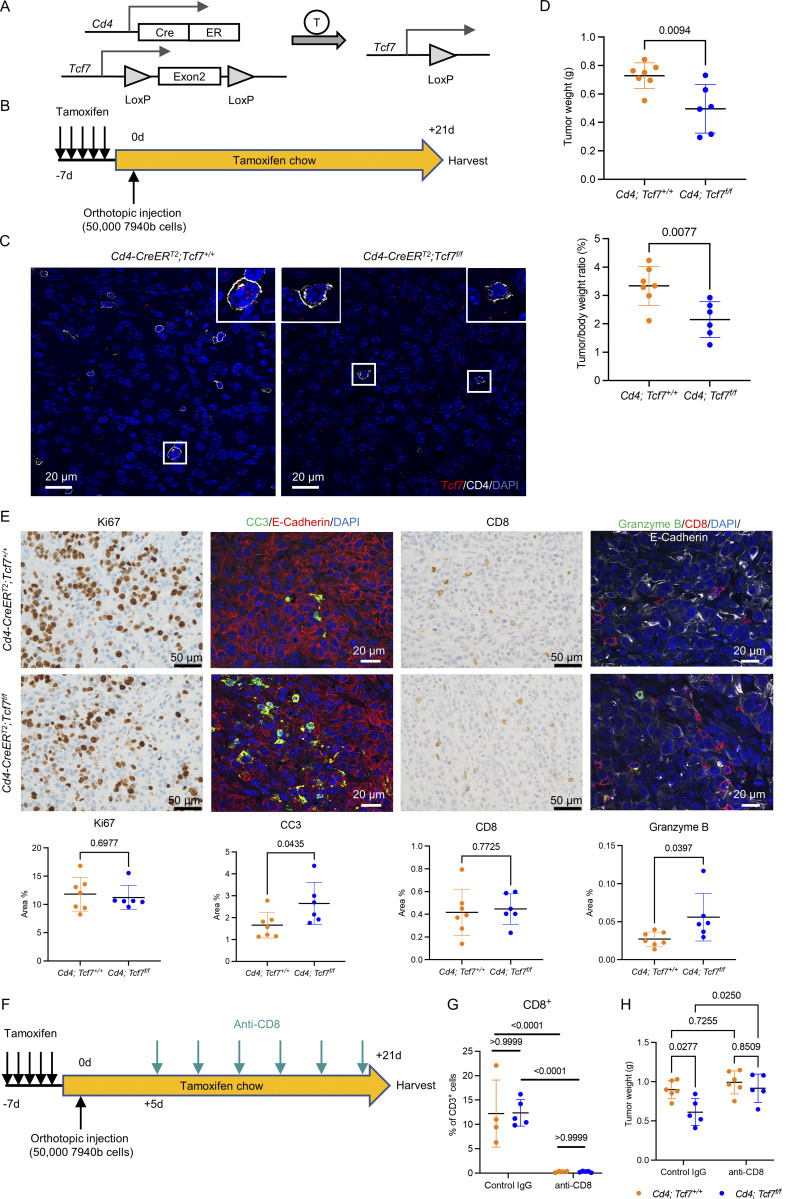
**TCF1 signaling in CD4**^**+**^ **T cells mediates their immunosuppressive potential. (A)** Genetic makeup of the *Cd4*;*Tcf7*^*fl/fl*^ mouse model. T = tamoxifen. **(B)** Experimental design. Tamoxifen was given to *Cd4*;*Tcf7*^*fl/fl*^ and *Cd4*;*Tcf7*^*+*^^/^^*+*^ mice in 5 consecutive days to deplete *Tcf7* in CD4^+^ T cells. The mice were then on tamoxifen chow until the end of the experiment. 5 × 10^4^ 7940b cells were injected into the pancreas of the mice. The experiment was independently performed twice. **(C)** RNA ISH of *Tcf7* (red) and co-IF of CD4 (white) and DAPI (blue) in the 7940b tumor tissues. Scale bar, 20 μm. **(D)** Final tumor weight and tumor-to–body weight ratio in *Cd4*;*Tcf7*^*fl/fl*^ and *Cd4*;*Tcf7*^*+*^^/^^*+*^ mice (*n* = 6–7/group). **(E)** Immunostaining of indicated markers. CC3 = cleaved caspase 3. Scale bar, 50 μm for IHC and 20 μm for co-IF. Quantification is shown at the bottom (*n* = 6–7/group). **(F)** Experimental design of CD8^+^ T cell depletion. **(G)** CD3^+^ CD8^+^ T cells in the 7940b tumors from *Cd4*;*Tcf7*^*fl/fl*^ and *Cd4*;*Tcf7*^*+*^^/^^*+*^ mice treated with control IgG or anti-CD8 antibody were measured by flow cytometry as a percentage of CD3^+^ T cells (*n* = 4–5/group). **(H)** Final tumor weight in *Cd4*;*Tcf7*^*fl/fl*^ and *Cd4*;*Tcf7*^*+*^^/^^*+*^ mice treated with control IgG or anti-CD8 antibody (*n* = 5–6/group). Two-tailed Student *t* test (two groups) and one-way ANOVA with Tukey test (four groups) were performed for comparison between groups. P < 0.05 was considered significant.

**Figure S2.**
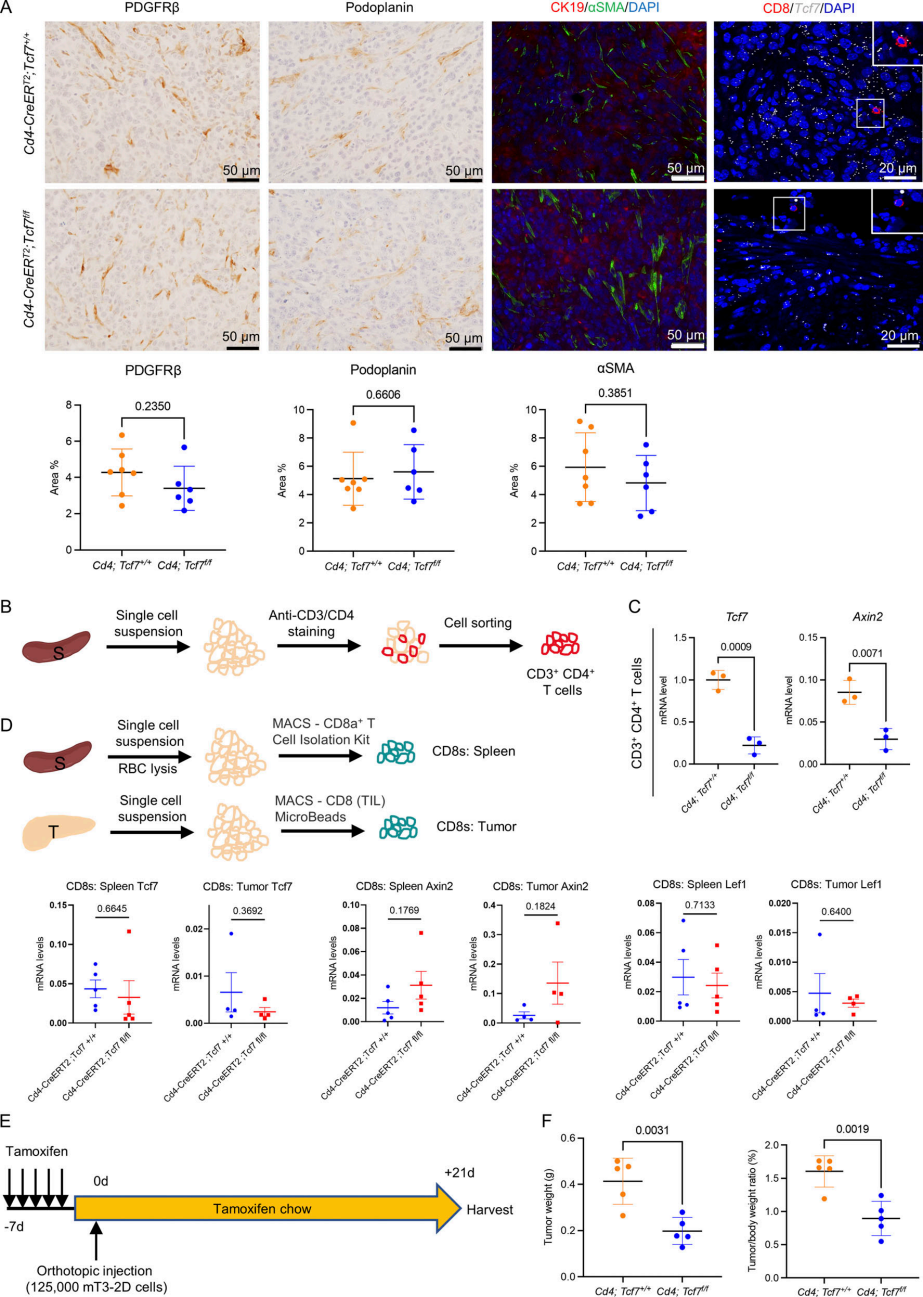
**TCF1 in CD4**^**+**^ **T cells promotes pancreatic cancer growth. (A)** Immunostaining of indicated markers in the 7940b tumor tissues. Scale bar, 20 or 50 μm. Quantification is shown at the bottom (*n* = 6–7/group). **(B)** Schematic illustration of splenic CD4^+^ T cells extraction by FACS. S = spleen; T = tumor. **(C)** qRT-PCR for *Tcf7* and *Axin2* expression in splenic CD4^+^ T cells derived from tumor-bearing *Cd4*;*Tcf7*^*fl/fl*^ and *Cd4*;*Tcf7*^*+*^^/^^*+*^ mice (*n* = 3/group). **(D)** qRT-PCR for *Tcf7*, *Lef1*, and *Axin2* expression in CD8^+^ T cells derived from tumor-bearing *Cd4*;*Tcf7*^*fl/fl*^ and *Cd4*;*Tcf7*^*+*^^/^^*+*^ mice (*n* = 4–5/group). **(E)** Experimental design. 1.25 × 10^5^ mT3-2D cells were injected into the pancreas of the mice. **(F)** Final tumor weight and tumor-to–body weight ratio in *Cd4*;*Tcf7*^*fl/fl*^ and *Cd4*;*Tcf7*^*+*^^/^^*+*^ mice (*n* = 5/group). Two-tailed Student *t* test (two groups) was performed for comparison between groups. P < 0.05 was considered significant.

To study the role of TCF1 in CD4^+^ T cells, we generated *Cd4-CreER*^*T2*^;*Tcf7*^*fl/fl*^ mice (referred to as *Cd4;Tcf7*^*fl/fl*^; Fig. 2 A). The mice were treated with tamoxifen to delete *Tcf7* specifically in CD4 expressing T cells and then injected with syngeneic 7940b KPC (*Kras*^*LSL-G12D*^; *Trp53*^*flox/+*^*; Ptf1a-Cre*) cells orthotopically in the pancreata to establish tumors (Fig. 2 B). 3 wk later, we harvested the tumors for analysis. Deletion of *Tcf7* in CD4^+^ T cells was confirmed by in situ hybridization of *Tcf7* together with immunofluorescence (IF) staining for CD4 (Fig. 2 C). As all T cells go through a double positive CD4^+^;CD8^+^ stage prior to differentiating into either CD4 or CD8 single positive cells (Germain, 2002), we then sought to determine whether CD8^+^ T cells had also lost *Tcf7* expression; however, doing a parallel RNA scope staining with the same probe, we detected no *Tcf7* expression in CD8^+^ T cells even in control tumors (Fig. S2 A). As an alternative approach, we sorted CD4^+^ T cells and CD8^+^ T cells from the spleens of tumor-bearing *Cd4*;*Tcf7*^*fl/fl*^ and control *Cd4-CreER*^*T2*^;*Tcf7*^*+/+*^ mice (referred to as *Cd4;Tcf7*^*+/+*^; Fig. S2 B) and used quantitative RT-PCR (qRT-PCR) to measure the expression levels of *Tcf7* and the WNT signaling target gene *Axin2*. As expected, CD4^+^ T cells from *Cd4*;*Tcf7*^*fl/fl*^ mice had lower expression of *Tcf7* and *Axin2* (Fig. S2 C). Basal expression of both genes was lower in CD8^+^ T cells in the control mice and did not significantly decrease in *Cd4*;*Tcf7*^*fl/fl*^ mice, nor did expression of the WNT target gene *Lef1*, possibly indicating low/no WNT signaling activity in this cell population (Fig. S2, C and D).

When we compared final tumor weight and tumor-to–body weight ratio, we noticed a significant reduction in tumor growth in *Cd4;Tcf7*^*fl/fl*^ mice (Fig. 2 D) compared with control hosts. We then repeated the orthotopic syngeneic transplantation experiment with the mT3-2D KPC cell line (Fig. S2 E) and again observed smaller tumors (tumor weight and tumor-to–body weight ratio) in *Cd4;Tcf7*^*fl/fl*^ mice compared with control hosts (Fig. S2 F).

To understand why the tumors were smaller in *Cd4;Tcf7*^*fl/fl*^ mice, we performed immunohistochemical (IHC) and co-IF analysis of the 7940b tumor tissues. The results revealed no change in cell proliferation (Ki67; Fig. 2 E) and fibroblast accumulation or activation (Fig. S2 A) but an increase in tumor cell apoptosis (measured by cleaved caspase 3; Fig. 2 E), indicating that the smaller tumor size in mice with *Tcf7* deletion in CD4^+^ T cells is probably due to enhanced epithelial cell death. We then evaluated the tumor-infiltrating CD8^+^ T cells. While we observed no change in the infiltration of CD8^+^ T cells, we noticed an increase in Granzyme B expression in *Cd4;Tcf7*^*fl/fl*^ mice (Fig. 2 E), indicating upregulated cytotoxic activity. This result is consistent with our previous finding that ablation of CD4^+^ T cells resulted in activation of CD8^+^ T cells (Zhang et al., 2020; Zhang et al., 2014), indicating that the immunosuppressive function of CD4^+^ T cells might be mediated by TCF1.

To establish whether CD8^+^ T cell cytotoxic activity is directly responsible for the reduction in tumor growth, we repeated the orthotopic syngeneic transplantation experiment with 7940b KPC cells in *Cd4;Tcf7*^*fl/fl*^ and *Cd4;Tcf7*^*+/+*^ mice, and randomized mice to receive isotype control IgG or anti-CD8 antibody (Fig. 2 F). Efficient depletion of CD8^+^ T cells was confirmed by flow cytometry analysis (Fig. 2 G). Tumor growth was rescued upon depletion of CD8^+^ T cells (Fig. 2 H), supporting a model whereby the reduction in tumor growth in *Cd4; Tcf7*^*fl/fl*^ mice was CD8^+^ T cell dependent.

### TCF1 signaling in CD4^+^ T cells promotes Treg differentiation in PDA

**Figure 3.**
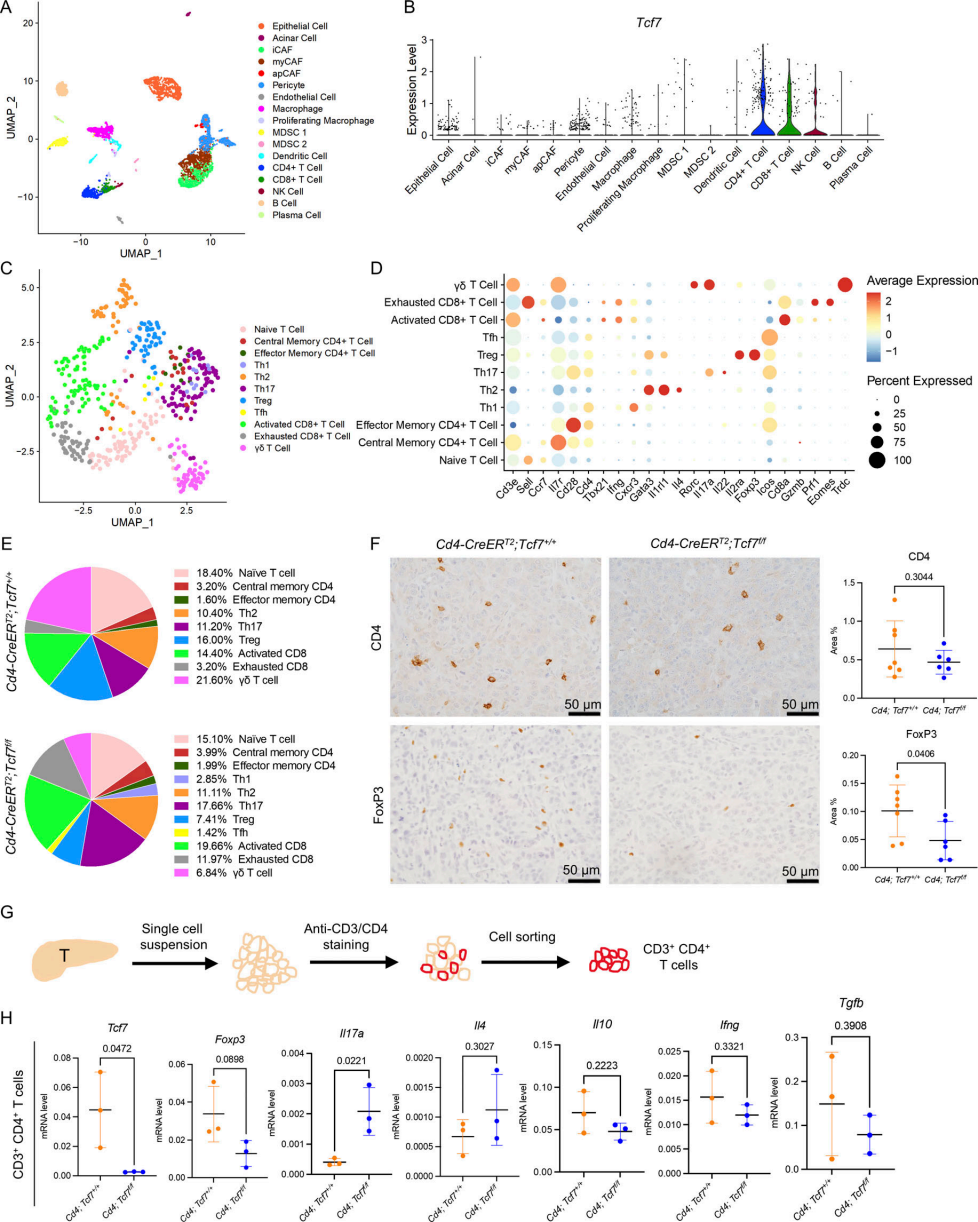
**TCF1 signaling in CD4**^**+**^ **T cells promotes Treg differentiation in PDA. (A)** UMAP representation of identified cell populations in mPDA orthotopic tumors. **(B)** Violin plot of *Tcf7* in all the identified cell populations. **(C)** UMAP representation of identified T cell populations in mPDA orthotopic tumors. **(D)** Dot plot of the key markers used to define the identified cell populations. **(E)** Percentage of individually identified cell populations in total T cells. **(F)** IHC of CD4 and FoxP3 in the 7940b tumor tissues. Scale bar, 50 μm. Quantification is shown on the right (*n* = 6–7/group). **(G)** Schematic illustration of CD4^+^ T cell extraction by FACS. T = tumor. **(H)** qRT-PCR for *Tcf7*, *Foxp3*, *Il17a*, *Il4*, *Il10*, *Tgfb*, and *Ifng* expression in CD4^+^ T cells derived from tumor-bearing *Cd4*;*Tcf7*^*fl/fl*^ and *Cd4*;*Tcf7*^*+*^^/^^*+*^ mice (*n* = 3/group). Two-tailed Student *t*-test (two groups) was performed for comparison between groups. P < 0.05 was considered significant. iCAF, inflammatory cancer associated fibroblasts; myCAF, myofibroblastic cancer associated fibroblasts; apCAF, antigen-presenting cancer associated fibroblasts.

**Figure S3.**
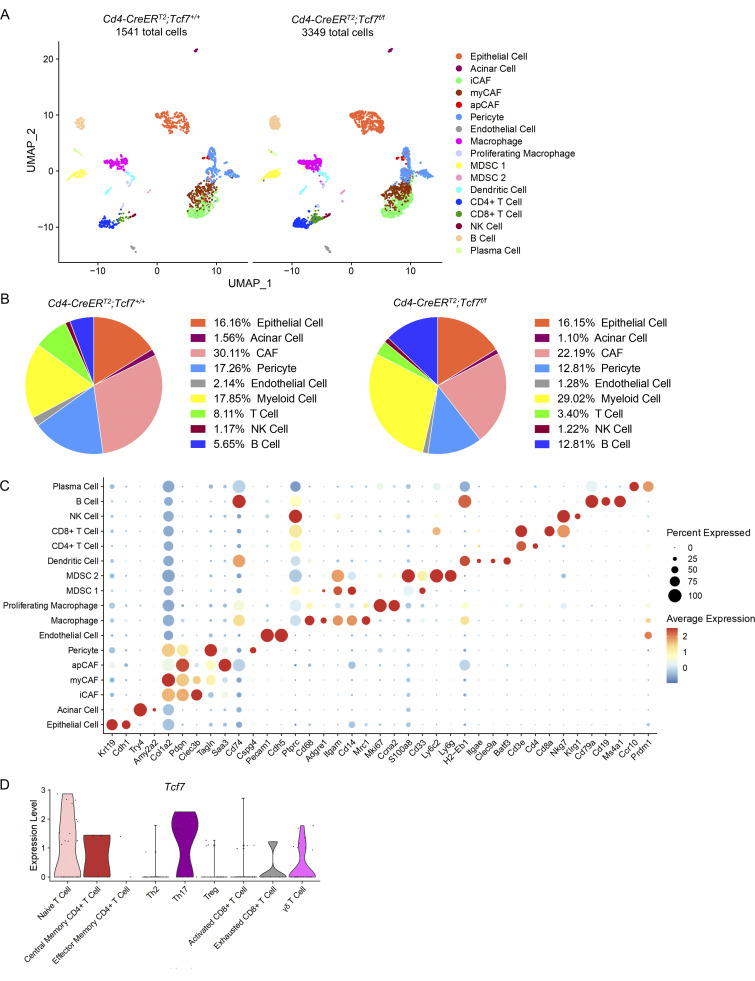
**scRNA-seq data of mPDA orthotopic tumors. (A)** UMAP representation of identified cell populations in mPDA orthotopic tumors. **(B)** Percentage of individual identified cell populations in total cells. **(C)** Dot plot of the key markers used to define the identified cell populations. **(D)** Violin plot of *Tcf7* in identified T cell populations from *Cd4;Tcf7*^*+*^^/^^*+*^ mice. iCAF, inflammatory cancer associated fibroblasts; myCAF, myofibroblastic cancer associated fibroblasts; apCAF, antigen-presenting cancer associated fibroblasts.

To investigate the immune microenvironment, we generated scRNA-seq data from 7940b tumors in *Cd4;Tcf7*^*fl/fl*^ and *Cd4;Tcf7*^*+/+*^ mice. We identified different populations by unbiased clustering and visualization using the UMAP algorithm (Fig. 3 A; and Fig. S3, A and B). Cell identities for each cluster were determined based on expression of known lineage markers (Fig. S3 C). We confirmed the expression of *Tcf7* in T cells and NK cells, with the highest expression in CD4^+^ T cells in this orthotopic model (Fig. 3 B). To explore the changes in T cells upon *Tcf7* inactivation, we performed unbiased subclustering of *Cd3e* expressing cells and identified 11 distinct populations (Fig. 3, C and D). The expression of *Tcf7* was high in naive T cells, central memory CD4^+^ T cells, and T helper 17 (Th17) cells in *Cd4;Tcf7*^*+/+*^ tumors (Fig. S3 D). The percentages of naive T cells, memory CD4^+^ T cells, and T helper 2 (Th2) cells were similar in *Cd4;Tcf7*^*fl/fl*^ and *Cd4;Tcf7*^*+/+*^ mice (Fig. 3 E). Given the paucity of T helper 1 (Th1) cells and T follicular helper (Tfh) cells in the TME, they were not detected in *Cd4;Tcf7*^*+/+*^ mice and only comprised 2.85% (Th1) and 1.42% (Tfh) of T cells in *Cd4;Tcf7*^*fl/fl*^ mice (Fig. 3 E). Increased percentages of activated CD8^+^ T cells (19.66 vs. 14.40%) and exhausted CD8^+^ T cells (11.97 vs. 3.2%) were observed in *Cd4;Tcf7*^*fl/fl*^ mice compared with *Cd4;Tcf7*^*+/+*^ mice (Fig. 3 E). Interestingly, tumors from *Cd4;Tcf7*^*fl/fl*^ mice also presented with a relative increase in Th17 cells (17.66 vs. 11.20%) and, conversely, a decreased percentage of Tregs (7.41 vs. 16.00%) and γδ T cells (6.84 vs. 21.6%; Fig. 3 E).

To validate changes observed in scRNA-seq data, we performed IHC analysis on 7940b tumors. Consistent with the sequencing data, we observed no difference in CD4^+^ T cell infiltration but a reduction in FoxP3 staining, indicating decreased Tregs (Fig. 3 F). To further characterize TCF1-null CD4^+^ T cells, we sorted CD4^+^ T cells from 7940b tumors in *Cd4;Tcf7*^*fl/fl*^ or control mice (Fig. 3 G). We then used qRT-PCR to characterize CD4^+^ T cells (Fig. 3 H). As expected, we observed a decrease in *Tcf7* consistent with efficient recombination of the locus. We also observed reduced *Foxp3* expression and, conversely, an increase in *Il17a* expression in the sorted CD4^+^ T cells from *Cd4;Tcf7*^*fl/fl*^ mice. Other cytokines, including the Th1 cytokine *Ifng,* the Th2 cytokine *Il4*, and Treg markers *Il10* and *Tgfb* showed no significant change. Taken together, our data show that inactivation of *Tcf7* in CD4^+^ T cells reduces Treg differentiation and promotes Th17 cell fate.

### Loss of TCF1 in CD4^+^ T cells leads to a compensatory increase in MDSCs

**Figure 4.**
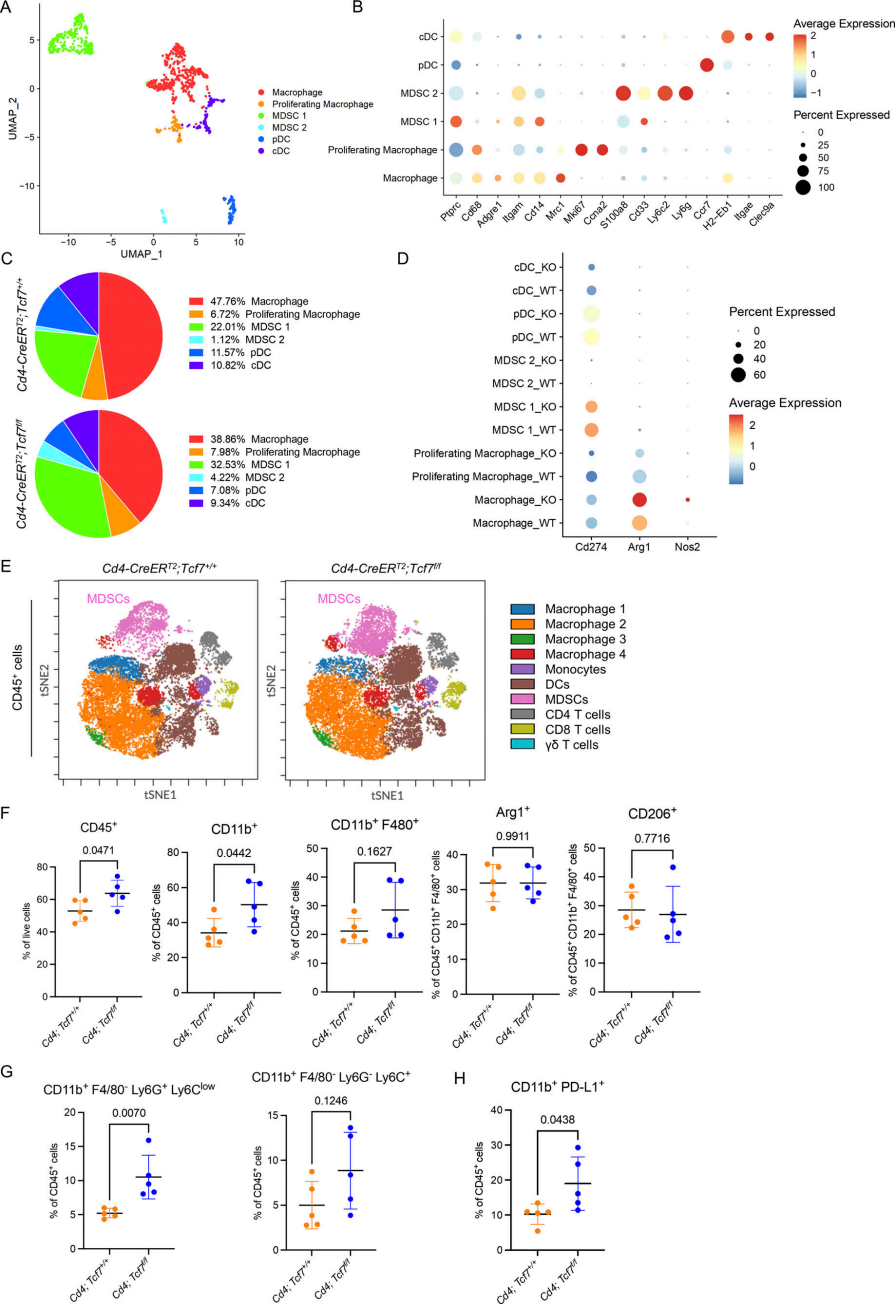
**Loss of TCF1 in CD4**^**+**^ **T cells leads to a compensatory increase in MDSCs. (A)** UMAP representation of identified myeloid cell populations in mPDA orthotopic tumors. **(B)** Dot plot of the key markers used to define the identified cell populations. **(C)** Percentage of individually identified cell populations in total myeloid cells. **(D)** Dot plot of *Cd274*, *Arg1*, and *Nos2* in all the identified cell populations in tumors from *Cd4*;*Tcf7*^*fl/fl*^ and *Cd4*;*Tcf7*^*+*^^/^^*+*^ mice. WT = *Cd4*;*Tcf7*^*+*^^/^^*+*^; KO = *Cd4*;*Tcf7*^*fl/fl*^. cDC = conventional dendritic cell; pDC = plasmacytoid dendritic cell. **(E)** Representative t-distributed stochastic neighbor embedding (tSNE) visualization of immune cell populations identified using CyTOF in 7940b tumors. **(F)** Manual gating quantitation of myeloid cell populations in 7940b tumors (*n* = 5/group). Populations include total immune cells (CD45^+^), total myeloid cells (CD45^+^ CD11b^+^), macrophages (CD45^+^ CD11b^+^ F4/80^+^), Arg1^+^ macrophages, and CD206^+^ macrophages. **(G)** Manual gating quantitation of Gr-MDSCs (CD11b^+^ F4/80^−^ Ly-6C^low^ Ly6G^+^) and M-MDSCs (CD11b^+^ F4/80^−^ Ly-6C^+^ Ly-6G^−^) in 7940b tumors (*n* = 5/group). **(H)** Manual gating quantitation of PD-L1^+^ CD11b^+^ myeloid cells in 7940b tumors (*n* = 5/group). Two-tailed Student *t*-test (two groups) was performed for comparison between groups. P < 0.05 was considered significant.

To determine whether inactivation of *Tcf7* in CD4^+^ T cells altered the composition of myeloid cells within the PDA TME, we performed unbiased subclustering of myeloid cells from the scRNA-seq data. We identified six distinct populations, including macrophages, proliferating macrophages, MDSC 1 and MDSC 2, plasmacytoid dendritic cells, and conventional dendritic cells (Fig. 4, A and B). The percentages of both MDSC 1 (32.53 vs. 22.01%) and MDSC 2 (4.22 vs. 1.12%) were higher in *Cd4;Tcf7*^*fl/fl*^ mice compared with *Cd4;Tcf7*^*+/+*^ mice (Fig. 4 C). Conversely, the percentages of macrophages and dendritic cells dropped in *Cd4;Tcf7*^*fl/fl*^ mice (Fig. 4 C). We then investigated the immunosuppressive potential of the myeloid cells. Programmed cell death ligand 1 (PD-L1), encoded by *Cd274*, acts as a co-inhibitory factor of the immune response. It can bind to its receptor programmed cell death protein 1 (PD-1) on activated T cells to inhibit their cytokine secretion ability and induce apoptosis (Chen, 2004; Zou and Chen, 2008). However, different subpopulations of myeloid cells had similar *Cd274* expression levels between two genotypes (Fig. 4 D). While the relative abundance of myeloid cells that express *Arg1*^*+*^ and *Nos2*^+^ did not change, the levels of expression per cell of both markers were higher in *Cd4;Tcf7*^*fl/fl*^ mice (Fig. 4 D), suggesting an increased immunosuppressive phenotype (Bhatt et al., 2014; Ellyard et al., 2010). Intriguingly, these data are consistent with previous observations from our group, showing an increase in immature myeloid cells and an increase in *Arg1* expression upon CD4^+^ T cell or Treg depletion in PanINs (Zhang et al., 2020).

**Figure S4.**
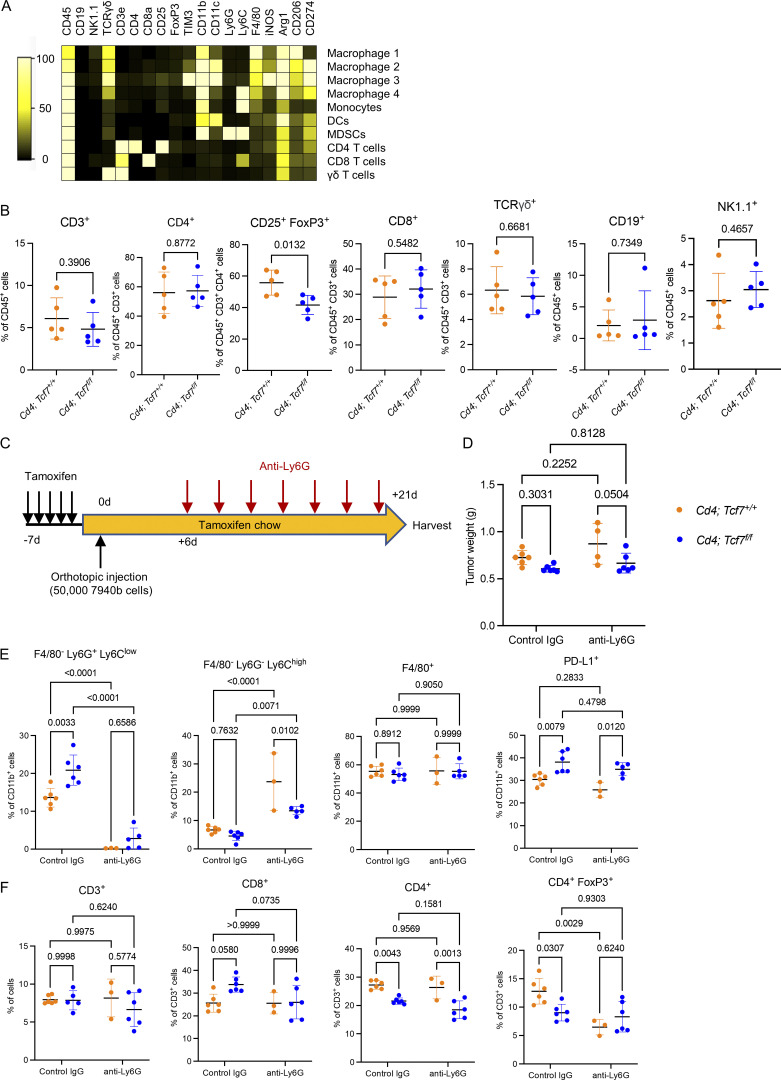
**Gr-MDSC depletion failed to improve the antitumor effect of TCF1 loss in CD4**^**+**^ **T cells. (A)** Markers used to identify immune cell populations in CyTOF. **(B)** Manual gating quantitation of immune cell populations in 7940b tumors (*n* = 5/group). Populations include total T cells (CD45^+^ CD3^+^), CD4 T cells (CD3^+^ CD4^+^), Tregs (CD4^+^ CD25^+^ FoxP3^+^), CD8 T cells (CD3^+^ CD8^+^), γδ T cells (CD3^+^ TCRγδ^+^), B cells (CD45^+^ CD19^+^), and NK cells (CD45^+^ NK1.1^+^). **(C)** Experimental design of Ly6G^+^ MDSC depletion. **(D)** Final tumor weight in *Cd4*;*Tcf7*^*fl/fl*^ and *Cd4*;*Tcf7*^*+*^^/^^*+*^ mice treated with control IgG or anti-Ly6G antibody (*n* = 4–6/group). **(E)** Gr-MDSCs (CD11b^+^ F4/80^−^ Ly-6C^low^ Ly6G^+^), M-MDSCs (CD11b^+^ F4/80^−^ Ly-6C^+^ Ly-6G^−^), macrophages (CD11b^+^ F4/80^+^), and PD-L1^+^ CD11b^+^ myeloid cells in the 7940b tumors treated with control IgG or anti-Ly6G antibody were measured by flow cytometry as a percentage of CD11b^+^ myeloid cells (*n* = 3–6/group). **(F)** CD3^+^ T cells, CD3^+^ CD8^+^ T cells, CD3^+^ CD4^+^ T cells, and CD3^+^ CD4^+^ FoxP3^+^ T cells were measured by flow cytometry as a percentage of total cells or CD3^+^ T cells (*n* = 3–6/group). Two-tailed Student *t* test (two groups) and one-way ANOVA with Tukey test (four groups) were performed for comparison between groups. P < 0.05 was considered significant.

MDSCs consist of two major subsets, monocytic-MDSCs (M-MDSCs) and granulocytic-MDSCs (Gr-MDSCs), which are distinguished based on the expression level of Ly-6G and Ly-6C. To complement our sequencing approach and identify which subpopulation of MDSCs increased upon *Tcf7* depletion in CD4^+^ T cells, we performed cytometry by time-of-flight (CyTOF) analysis of 7940b tumors from *Cd4;Tcf7*^*fl/fl*^ and *Cd4;Tcf7*^*+/+*^ mice. Unbiased clustering of CD45^+^ immune cells visualized through FlowSOM-viSNE revealed heterogeneous immune populations (Fig. 4 E and Fig. S4 A). *Cd4;Tcf7*^*fl/fl*^ mice had increased total immune cells (CD45^+^) as proportion of total live cells (Fig. 4 F). Manual gating showed a lower proportion of Tregs (CD45^+^ CD3^+^ CD4^+^ CD25^+^ FoxP3^+^) among CD4^+^ T cells in *Cd4;Tcf7*^*fl/fl*^ mice (Fig. S4 B). Conversely, we observed no significant difference in total T cells (CD45^+^ CD3^+^), CD4^+^ T cells (CD45^+^ CD3^+^ CD4^+^), CD8^+^ T cells (CD45^+^ CD3^+^ CD8^+^), B cells (CD45^+^ CD19^+^), NK cells (CD45^+^ NK1.1^+^), and γδ T cells (CD45^+^ CD3^+^ TCRγδ^+^) in *Cd4;Tcf7*^*fl/fl*^ mice (Fig. S4 B). Total myeloid cells (CD45^+^ CD11b^+^) were increased in *Cd4;Tcf7*^*fl/fl*^ mice, but we observed no significant change in the number and polarization of macrophages (CD45^+^ CD11b^+^ F4/80^+^) or M-MDSCs (CD45^+^ CD11b^+^ F4/80^−^ Ly-6C^+^ Ly-6G^−^; Fig. 4, F and G). We found that the increase in myeloid cells was due largely to an increase in Gr-MDSCs (CD45^+^ CD11b^+^ F4/80^−^ Ly-6C^low^ Ly6G^+^; Fig. 4 G). Further, we observed an increase in PD-L1^+^ myeloid cells (CD45^+^ CD11b^+^ PD-L1^+^) in *Cd4;Tcf7*^*fl/fl*^ mice (Fig. 4 H). These data are consistent with a compensatory immunosuppressive response driven by myeloid cells, following loss of TCF1 in CD4^+^ T cells, as shown by scRNA-seq analysis.

Given the increase in Gr-MDSCs, we hypothesized that they might mediate residual immune suppression, preventing a complete tumor-inhibitory CD8^+^ T cell response in our *Cd4;Tcf7*^*fl/fl*^ mice. To test this possibility, we repeated the orthotopic syngeneic transplantation experiment as described above and randomized the mice to receive isotype control IgG or anti-Ly6G antibody, which depletes Gr-MDSCs (Stromnes et al., 2014; Fig. S4 C). Our results showed no synergy between anti-Ly6G and *Tcf7* deletion in this model (Fig. S4 D), although efficient Gr-MDSC depletion was obtained (Fig. S4 E). We then investigated changes in immune composition upon treatment with anti-Ly6G in *Cd4;Tcf7*^*fl/fl*^ and *Cd4;Tcf7*^*+/+*^ mice. Both in *Cd4;Tcf7*^*fl/fl*^ and *Cd4;Tcf7*^*+/+*^ mice, depletion of Ly6G^+^ cells resulted in a large increase in M-MDSCs, possibly compensating for the lack of Gr-MDSCs. This may explain why anti-Ly6G did not have an effect on tumor growth, even as MDSCs were previously described as mediators of immunosuppression in pancreatic cancer (Stromnes et al., 2014). While we observed no changes in infiltrating CD8^+^ T cells, anti-Ly6G treatment resulted in a reduction in CD4^+^ T cells, largely explained by Tregs, but the extent of the reduction was lesser than what was observed upon loss of *Tcf7* in CD4^+^ T cells (Fig. S4 F).

Taken together, our data showed no benefit in depleting Gr-MDSCs in the context of ablation of TCF1 signaling in CD4^+^ T cells. In consequence, we shifted our focus to investigate the increase in PD-L1^+^ cells in *Cd4;Tcf7*^*fl/fl*^ mice (Fig. S4 E) and to understand whether this finding could be exploited therapeutically.

### TCF1 depletion in CD4^+^ T cells drives PD-L1 expression in myeloid cells and tumor cells

**Figure 5.**
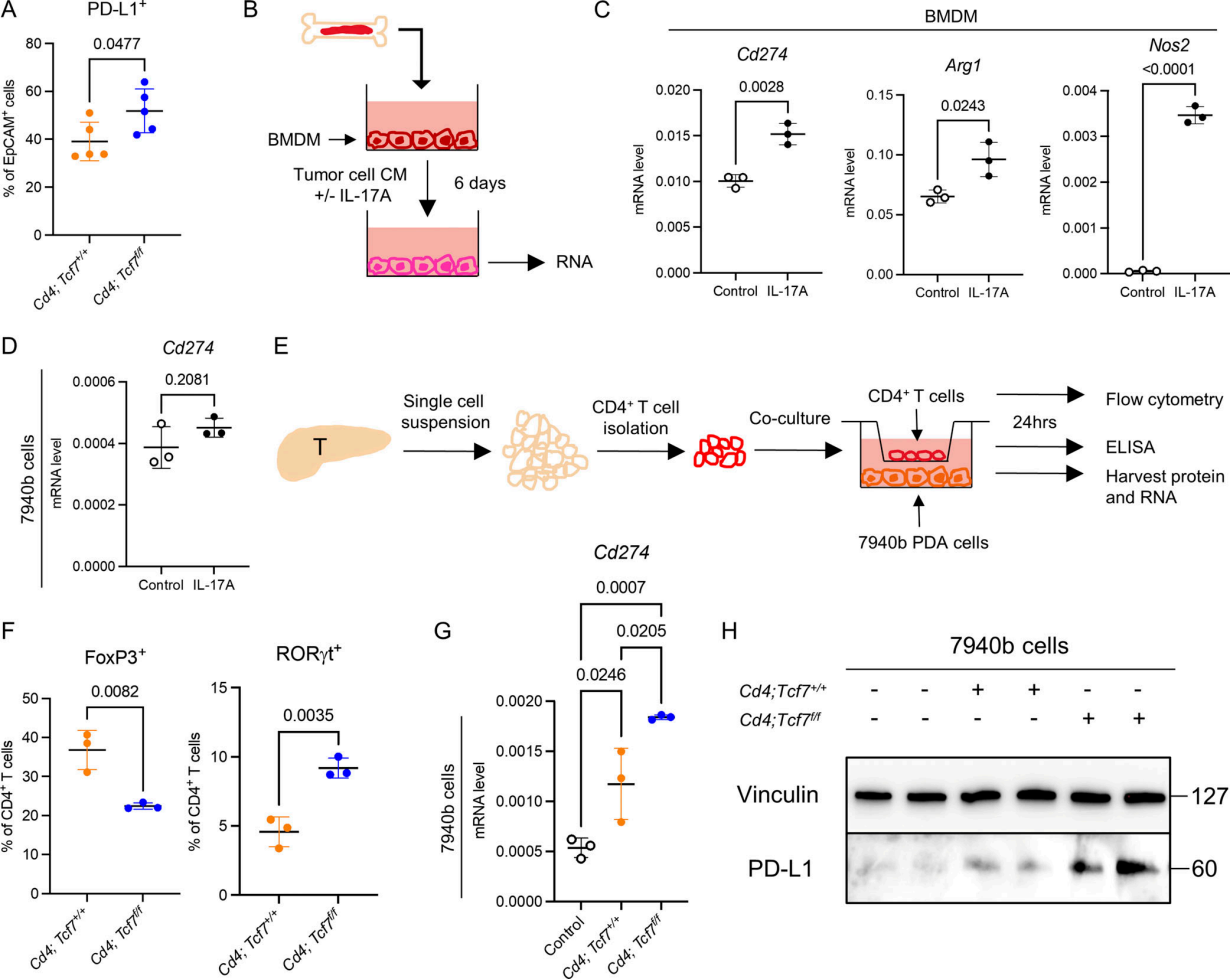
**TCF1 depletion in CD4**^**+**^ **T cells drives PD-L1 expression in myeloid cells and tumor cells. (A)** PD-L1^+^ EpCAM^+^ cells in the 7940b tumors were measured by CyTOF as a percentage of EpCAM^+^ cells (*n* = 5/group). **(B)** Experimental design of BMDM isolation and IL-17A treatment. The experiment was independently performed twice. CM = conditioned medium. **(C)** qRT-PCR for *Cd274*, *Arg1*, and *Nos2* expression in BMDM after IL-17A treatment (*n* = 3/group). **(D)** qRT-PCR for *Cd274* expression in 7940b cells treated with IL-17A for 24 h. **(E)** Experimental design of CD4^+^ T cell isolation and co-culture with epithelial cells. The experiment was independently performed twice. T = tumor. **(F)** FoxP3^+^ and RORγt^+^ CD4^+^ T cells after co-culture were measured by flow cytometry as a percentage of CD4^+^ T cells (*n* = 3/group). **(G)** qRT-PCR for *Cd274* expression in 7940b cells after co-culture (*n* = 3/group). **(H)** Western blot for PD-L1 expression (Vinculin as loading control) in 7940b cells after co-culture. Molecular weight markers are in kD. Two-tailed Student *t* test (two groups) and one-way ANOVA with Tukey test (three groups) were performed for comparison between groups. P < 0.05 was considered significant. Source data are available for this figure: SourceData F5.

**Figure S5.**
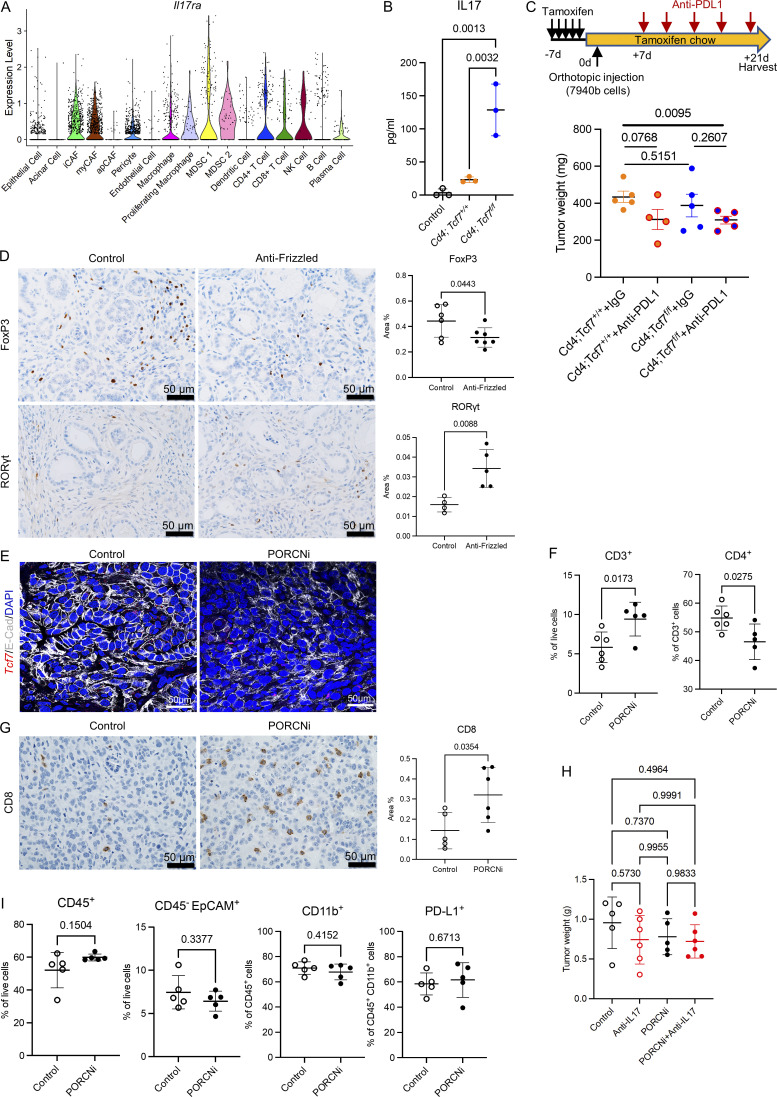
**Pharmacological WNT signaling increases CD8**^**+**^ **T cell infiltration. (A)** Violin plot of *Il17ra* in all the identified cell populations of mPDA orthotopic model. **(B)** The level of IL-17 in the medium after co-culture (*n* = 3/group). **(C)** Final tumor weight in *Cd4*;*Tcf7*^*fl/fl*^ and *Cd4*;*Tcf7*^*+*^^/^^*+*^ mice treated with control IgG or anti–PD-L1 antibody (*n* = 4–5/group). **(D)** IHC of FoxP3 and RORγt in the control or anti-FZD treated tissues. Scale bar, 50 μm. Quantification is shown on the right (*n* = 4–7/group). **(E)** Co-stain of *Tcf7*, E-cadherin, and DAPI in the tumors treated with control or PORCNi. Scale bar, 50 μm. **(F)** CD3^+^ T cells and CD3^+^ CD4^+^ T cells in the tumors treated with control or PORCNi were measured by flow cytometry (*n* = 5–6/group). **(G)** IHC of CD8 in the tumors treated with control or PORCNi. Scale bar, 50 μm. Quantification is shown on the right (*n* = 5–6/group). **(H)** Final tumor weight in mice treated with control or PORCNi or anti–IL-17 antibody or the combination (*n* = 5–6/group). **(I)** Total immune cells (CD45^+^), epithelial cells (CD45^−^ EpCAM^+^), total myeloid cells (CD45^+^ CD11b^+^), and PD-L1^+^ myeloid cells (CD45^+^ CD11b^+^ PD-L1^+^) in the tumors treated with control or PORCNi were measured by flow cytometry (*n* = 5–6/group). Two-tailed Student *t* test (two groups) and one-way ANOVA with Tukey test (three to four groups) were performed for comparison between groups. P < 0.05 was considered significant. iCAF, inflammatory cancer associated fibroblasts; myCAF, myofibroblastic cancer associated fibroblasts; apCAF, antigen-presenting cancer associated fibroblasts.

CyTOF analysis revealed an increase in PD-L1^+^ myeloid cells, as previously mentioned (Fig. S4 E), as well as increased PD-L1^+^ EpCAM^+^ cells in tumors from *Cd4*;*Tcf7*^*fl/fl*^ mice (Fig. 5 A). These tumors also had increased infiltration of Th17 cells, and previous literature linked IL-17 to PD-L1 expression in other cancer cells either alone or synergistically with IFN-γ/TNF-α (Wang et al., 2020; Wang et al., 2017; Wei et al., 2019). We noted that the IL-17 receptor gene (*Il17ra*) was expressed by epithelial cells and myeloid cells in our orthotopic model (Fig. S5 A). We thus tested whether IL-17 regulated PD-L1 expression in our system. We first isolated bone marrow–derived myeloid cells (BMDMs) from WT C57BL/6J mice and cultured them in tumor cell conditioned medium from 7940b cells with or without IL-17A (Fig. 5 B). We then collected RNA from the BMDMs after 6 d and measured the expression of *Cd274*, *Arg1*, and *Nos2* (Fig. 5, B and C). IL-17A induced an upregulation in the expression of all three genes in BMDMs (Fig. 5 C).

We then treated 7940b cells with IL-17A and measured the expression of *Cd274*. Unlike in BMDMs, IL-17A did not induce *Cd274* upregulation in tumor cells (Fig. 5 D). To investigate whether TCF1-depleted CD4^+^ T cells directly induced PD-L1 expression in tumor cells, we flow-sorted CD4^+^ T cells from tumors in *Cd4*;*Tcf7*^*fl/fl*^ and *Cd4*;*Tcf7*^*+/+*^ mice and co-cultured them with tumor cells using a transwell approach, allowing exchange of secreted factors but no cell–cell contact (Fig. 5 E). We collected the isolated CD4^+^ T cells after the co-culture and used flow cytometry to confirm that *Tcf7*-null CD4^+^ T cells consisted of fewer Tregs and more Th17 cells (Fig. 5 F), as expected based on in vivo results. We also detected a higher level of IL-17 in the medium from the co-culture including *Tcf7*-null CD4^+^ T cells and tumor cells, as measured by ELISA (Fig. S5 B). We then harvested RNA from 7940b cells and measured the expression of *Cd274*. We found that when co-cultured with CD4^+^ T cells from tumors in *Cd4*;*Tcf7*^*+/+*^ mice, 7940b cells expressed more *Cd274* compared with tumor cells cultured alone (Fig. 5 G). Its expression was further induced by CD4^+^ T cells from tumors in *Cd4*;*Tcf7*^*fl/fl*^ mice (Fig. 5 G). We also harvested protein from the co-cultured 7940b cells and validated the increase in PD-L1 at the protein level by a Western blot. Corresponding to RNA expression results, CD4^+^ T cell co-culture induced PD-L1 in tumor cells, and the induction was further upregulated in presence of *Cd4*;*Tcf7*^*fl/fl*^ T cells (Fig. 5 H). Thus, secreted factors from TCF1-depleted CD4^+^ T cells directly induced PD-L1 expression in tumor cells. To investigate whether *Cd4*;*Tcf7*^*fl/fl*^ mice were more sensitive to anti–PD-L1 treatment than *Cd4*;*Tcf7*^*+/+*^ mice, we repeated the orthotopic syngeneic transplantation experiment with 7940b KPC cells in *Cd4;Tcf7*^*fl/fl*^ and *Cd4;Tcf7*^*+/+*^ mice, and randomized mice to receive isotype control IgG or anti–PD-L1 antibody. Tumor growth was similar in *Cd4;Tcf7*^*fl/fl*^ mice treated with anti–PD-L1 antibody compared with *Cd4;Tcf7*^*+/+*^ mice on the same treatment (Fig. S5 C), possibly indicating that other compensatory mechanisms limit antitumor responses in this model.

### Pharmacological WNT signaling inhibition improves the efficacy of PD-L1 blockade

Our data so far shows that, in addition to the previously known tumor-supportive role of WNT signaling in epithelial cells (Jiang et al., 2014; Pai et al., 2016; Sano et al., 2016; Zhang et al., 2013), its function in CD4^+^ T cells contributes to pancreatic cancer growth, at least in part through promoting immune suppression. With both facets of WNT signaling in mind, we next queried whether global interruption of WNT signaling in all cell compartments via pharmacological inhibition might similarly regulate the tumor immune microenvironment. We first analyzed tissues from KC mice treated with the anti-FZD antibody Vantictumab (Zhang et al., 2013). Immunostaining revealed a reduction in FoxP3 expression and an increase in RORγt expression in anti-FZD-treated tissues, consistent with a shift from Tregs to Th17 cells, and resembling the changes observed in *Cd4*;*Tcf7*^*fl/fl*^ mice (Fig. S5 D). These data might be explained as a direct effect of inhibiting WNT in T cells, as an indirect effect of systemic WNT inhibition, or by a combination of those factors.

**Figure 6.**
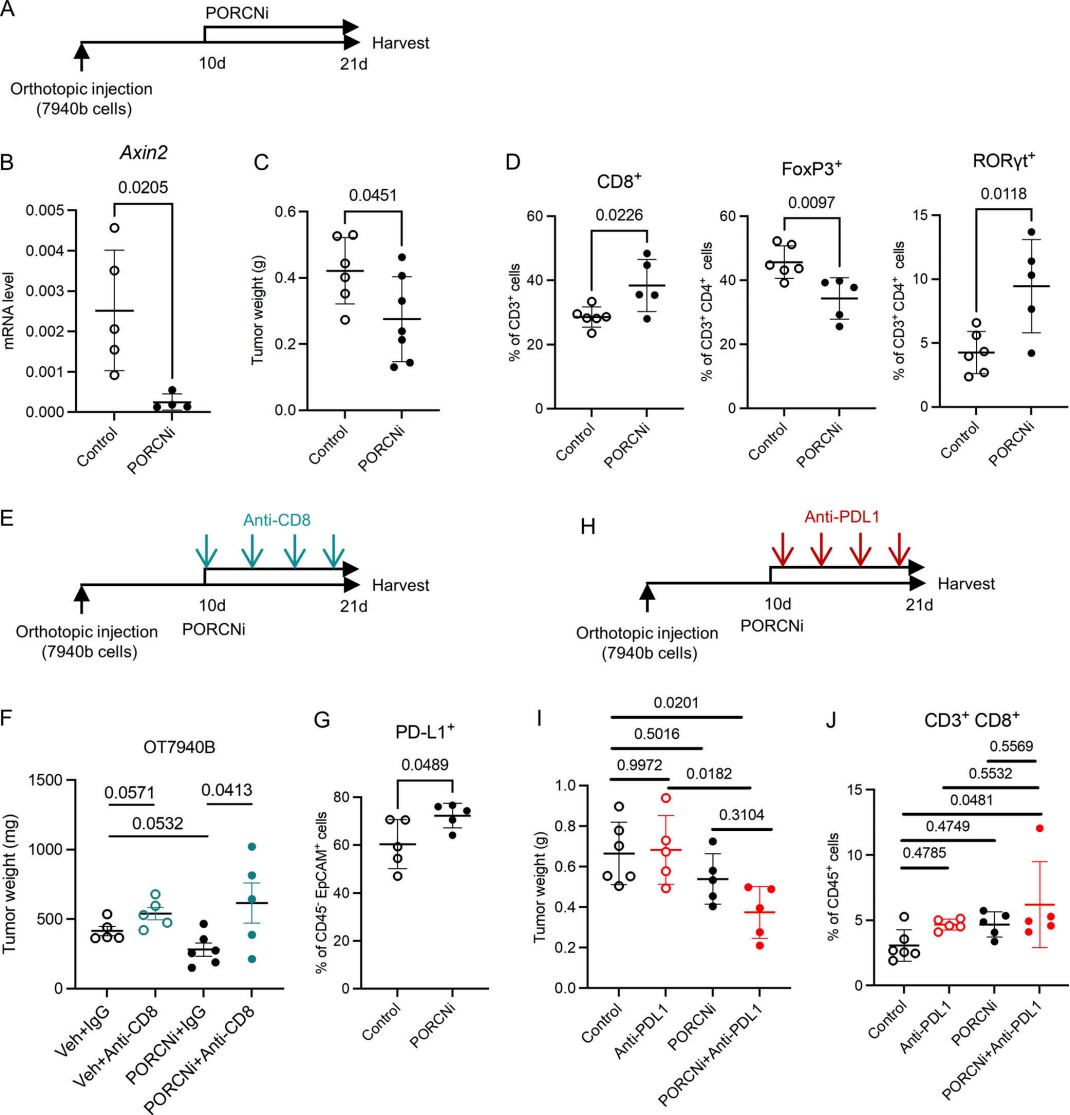
**Pharmacological WNT signaling inhibition improves the efficacy of PD-L1 blockade. (A)** Experimental design of pharmacological PORCN inhibition. **(B)** qRT-PCR for *Axin2* expression in tumors from mice treated with control or PORCNi (*n* = 4–5/group). **(C)** Final tumor weight in mice treated with control or PORCNi (*n* = 6–7/group). Each experiment was independently performed at least twice. **(D)** CD3^+^ CD8^+^ T cells, CD3^+^ CD4^+^ FoxP3^+^ T cells, and CD3^+^ CD4^+^ RORγt^+^ T cells were measured by flow cytometry (*n* = 5–6/group). **(E)** Experimental design of pharmacological PORCN inhibition in the combination with CD8 depletion antibody. **(F)** Final tumor weight in mice treated with control or PORCNi or anti-CD8 antibody or the combination (*n* = 5/group). **(G)** CD45^−^ EpCAM^+^ PD-L1^+^ cells were measured by flow cytometry (*n* = 5–6/group). **(H)** Experimental design of pharmacological PORCN inhibition in combination with PD-L1 blockade. **(I)** Final tumor weight in mice treated with control or PORCNi or anti–PD-L1 antibody or the combination (*n* = 5–6/group). **(J)** CD3^+^ CD8^+^ T cells were measured by flow cytometry as a percentage of CD3^+^ T cells (*n* = 5–6/group). Two-tailed Student *t* test (two groups) and one-way ANOVA with Tukey test (four groups) were performed for comparison between groups. P < 0.05 was considered significant.

We then used LGK974, a potent and specific small-molecule PORCN inhibitor (PORCNi; Liu et al., 2013), which inhibits ligand-dependent WNT signaling and is being tested in clinical trials in WNT-dependent malignancies (NCT01351103). We repeated the orthotopic syngeneic mouse model with 7940b pancreatic cancer cells and then randomized the mice to receive vehicle control or PORCNi (Fig. 6 A). We validated pathway inhibition by measuring the expression of the WNT target gene *Axin2,* which was reduced upon PORCNi treatment (Fig. 6 B). Overall *Tcf7* (including epithelial and non-epithelial cells) was also downregulated upon PORCNi treatment (Fig. S5 E). PORCNi treated tumors were smaller, consistent with a tumor-promoting role of WNT signaling (Fig. 6 C). We then evaluated whether systemic WNT inhibition altered the immune complement of the tumors. We thus performed flow cytometry and observed an increase in total T cell and CD8^+^ T cell infiltration in PORCNi treated tumors (Fig. 6 D and Fig. S5 F). IHC analysis of the tissues confirmed the increase in CD8^+^ T cell infiltration (Fig. S5 G). We also observed a decrease in CD4^+^ T cells and FoxP3^+^ Tregs and an increase in RORγt^+^ Th17 cells upon PORCNi treatment, consistent with the changes observed in our genetic model (Fig. 6 D). To establish whether the increase in Th17 cells blunted the effect of PORCNi treatment, given the known protumor effect of Th17 cells and IL-17 (McAllister et al., 2014; Zhang et al., 2018), we repeated the orthotopic syngeneic transplantation experiment with 7940b KPC cells and randomized mice to receive control, PORCNi, anti–IL-17 antibody, or a combination, but observed no change in tumor growth (Fig. S5 H).

To determine whether the reduction in tumor growth was due, at least in part, to induction of antitumor immunity, in addition to the direct effect on tumor cells, we combined PORCNi treatment with CD8^+^ T cell depletion (Fig. 6 E). Tumor growth was rescued upon combination of PORCNi and CD8^+^ T cell depletion (Fig. 6 F), indicating that the reduction of PORCNi on tumor growth was CD8^+^ T cell dependent.

Together, our data, using both genetic and pharmacologic approaches, indicate that inhibition of WNT signaling causes a shift in the immune microenvironment, with an increase in CD8^+^ T cell–mediated immune responses. At the same time, we observed potential compensatory immunosuppressive feedback mechanisms in PORCNi-treated tumors. Flow cytometry analysis showed an increase in PD-L1^+^ EpCAM^+^ cells upon PORCNi treatment with no difference in total EpCAM^+^ cells, total immune cells, myeloid cells, and PD-L1^+^ myeloid cells (Fig. 6 G and Fig. S5 I). Hypothesizing that the increase in PD-L1 expression might cause resistance to PORCNi treatment, we repeated the orthotopic syngeneic transplantation experiment, this time randomizing the mice to four treatment arms: (1) control vehicle + isotype control IgG; (2) control vehicle + anti–PD-L1 antibody; (3) PORCNi + isotype control IgG; and (4) PORCNi + anti–PD-L1 antibody (Fig. 6 H). PD-L1 blockade had no effect on the tumors, as previously described (Evans et al., 2016; Feig et al., 2013; Zhang. et al., 2019a), and in accordance with the lack of efficacy of the PD-1/PD-L1 immune checkpoint blockade in human pancreatic cancer patients (Brahmer et al., 2012; Fig. 6 I). In contrast, combination treatment with PORCNi and anti–PD-L1 reduced tumor growth beyond either of the single agent therapies (Fig. 6 I). Further, while either PORCNi alone or anti–PD-L1 antibody alone promoted CD8^+^ T cell infiltration, the combination treatment resulted in the highest number of infiltrating CD8^+^ T cells, consistent with a reprogramming of the immune response (Fig. 6 J). Taken together, WNT signaling inhibition sensitizes pancreatic cancer to PD-L1 blockade and should be explored as a potential therapeutic strategy for PDA patients.

## Discussion

**Figure 7.**
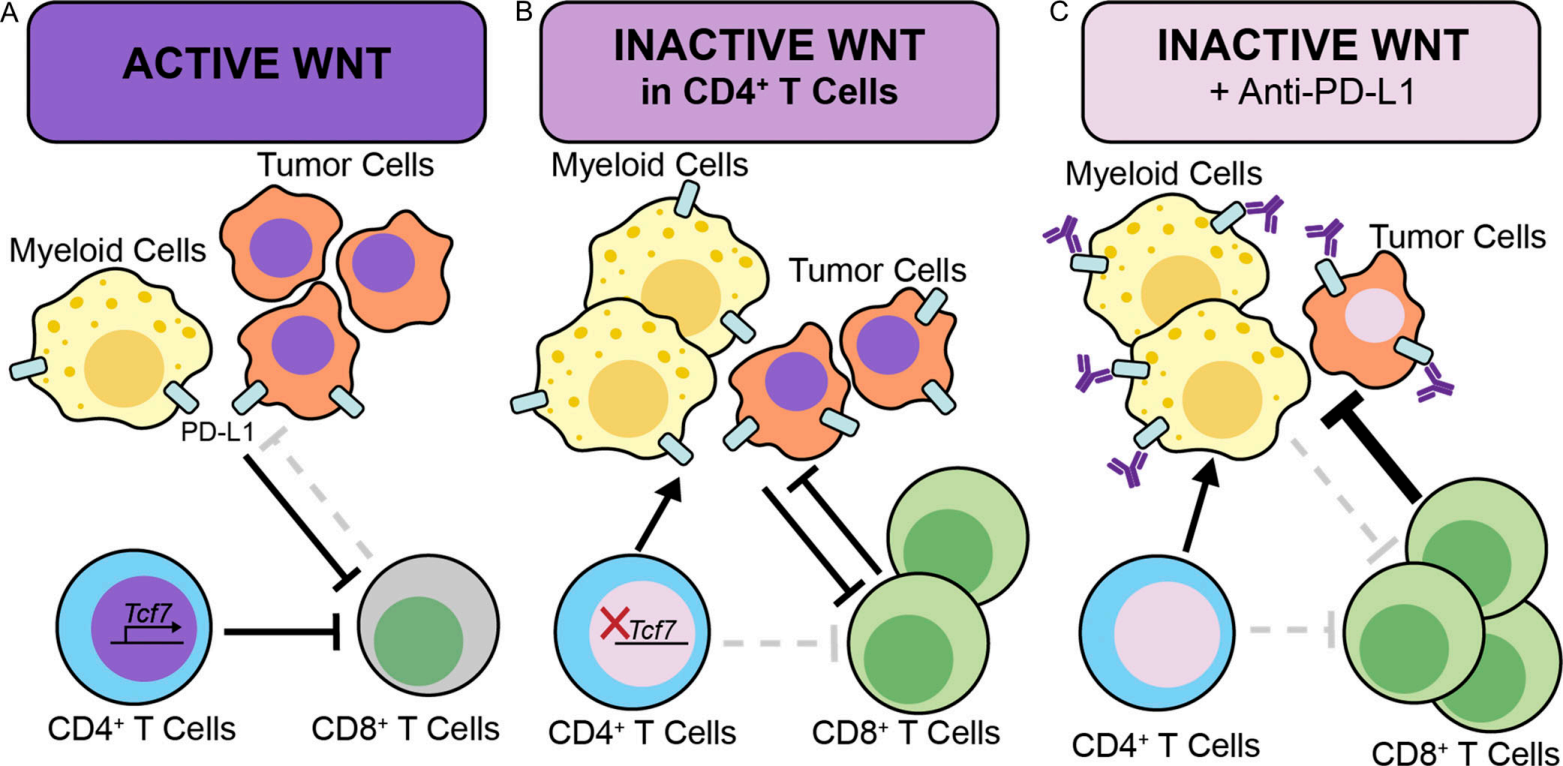
**Working model. (A)** WNT is active in epithelial cells and CD4^+^ T cells (purple nuclei) and contributes to blocking the antitumor activity of CD8^+^ T cells. **(B)** Inactivation of *Tcf7* in CD4^+^ T cells abrogates WNT signaling in a compartment-specific manner (pink nucleus) dampens tumor progression by enabling CD8^+^ T cell effector function. However, it also increases the number of PD-L1–expressing tumor and myeloid cells and leaves WNT signaling intact in tumor cells (purple nuclei). **(C)** Pharmacological inhibition abrogates WNT signaling in both tumor cells and CD4^+^ T cells, increasing the number of CD8^+^ T cells but also compensatory increase in PD-L1–expressing tumor cells. Combination of WNT inhibition and PD-L1 checkpoint blockade inhibit tumor growth though a mechanism that is, in part, immune mediated.

Pancreatic cancer is characterized by a complex signaling environment that mediates interactions between heterogeneous cell populations. Previously, we and others have linked reactivation of embryonic signaling pathways to pancreatic carcinogenesis (for review see Jones et al., 2008; Morris et al., 2010; Thayer et al., 2003). Among those, WNT signaling is activated through ligand overexpression or, in a subset of pancreatic cancer, through mutations in pathway components (Aguilera and Dawson, 2021; Deng et al., 2021; Jiang et al., 2013; Kim et al., 2021; Steinhart et al., 2017). Advances in single-cell technologies have allowed us to map these components in the pancreatic cancer microenvironment. We first validated expression of WNT components and target genes in human and mouse pancreatic cancer, supporting past work by our group on the functional importance of this pathway in epithelial cells (Zhang et al., 2013). Second, we discovered that WNT targets were also expressed in T cells, which also uniquely expressed the *TCF7* gene, encoding for TCF1. TCF1 is a known regulator of normal T cell development (Zhao et al., 2021) and has been studied in the context of CD8^+^ TCF1^+^ cells in cancers (Burger et al., 2021; Gattinoni et al., 2011; Han et al., 2021; Hanna et al., 2021; Sade-Feldman et al., 2018; Siddiqui et al., 2019). TCF1 has both WNT-dependent and -independent functions and has not been well studied in tumor-infiltrating CD4^+^ T cells. In colon cancer, a Treg population high in TCF1 promotes antitumor immunity (Osman et al., 2021). To address the functional role of TCF1 in CD4^+^ T cells in PDA TME, we generated mice where *Tcf7* could be inactivated upon Cre recombination in an inducible manner. We inactivated *Tcf7* in CD4^+^ T cells and implanted pancreatic cancer cells from the well-characterized KPC mouse model (Hingorani et al., 2005) into the pancreata of syngeneic C57BL/6J mice. We thus observed that tumors in mice lacking *Tcf7* in CD4^+^ T cells were smaller at endpoint. Interestingly, these tumors also had increased antitumor immunity mediated by activated CD8^+^ T cells (Fig. 7).

WNT inhibitors have been tested in clinical trials for multiple cancers including pancreatic cancer, but their therapeutic window is severely limited due to bone toxicity (Fischer et al., 2017; Kahn, 2014; Madan et al., 2018). For this reason, sustained WNT inhibition cannot be maintained in human patients. We reasoned that WNT inhibition could constitute a component of combination therapy. We first sought to determine whether systemic WNT inhibition would recapitulate the immune effects of TCF1 ablation in T cells. For this purpose, we analyzed archival tissues in our laboratory from KC mice (Hingorani et al., 2003) treated with OMP-185, a secreted FZD antagonist currently available under the name Vantictumab. Similar to the genetic model, we observed a reduction in Tregs. We then used a different WNT inhibitor, in this case LGK974, which blocks ligand-mediated WNT signaling by targeting PORCN and thus preventing ligand secretion. We then performed a detailed immune characterization of this model and noticed a reduction in Tregs and an increase in CD8^+^ T cells. Treatment of orthotopic syngeneic tumors with LGK974 reduced tumor growth, but compensatory mechanisms were evident. In particular, we observed an increase in PD-L1 expression. In several solid cancers, β-catenin induces transcriptional activation of PD-L1, while WNT inhibition results in decreased PD-L1 expression; those include breast (Castagnoli et al., 2019) and colon cancer (Ruan et al., 2020). However, here we observe an opposite effect, whereby PD-L1 is upregulated in cancer cells, both with T cell–specific loss of TCF1 and pharmacologic inhibition of WNT signaling, and these differences might reflect unique features of the pancreatic cancer microenvironment and highlight the need to study common signaling pathways in a cancer-type specific manner.

Mechanistically, we show that the increase in PD-L1 expression in tumor cells is directly induced by CD4^+^ T cells. While IL-17 controls PD-L1 expression in myeloid cells, it does not play any seeming role in driving PD-L1 expression in the tumor cells by itself. Regardless, the compensatory increase in PD-L1 supported the use of a combination approach. We thus treated mice bearing syngeneic orthotopic pancreatic cancer with either LGK974 alone, anti–PD-L1 alone, or a combination of the two and observed additive reduction in tumor growth.

Together, these findings set the stage for incorporating WNT inhibition as a component of pancreatic cancer therapy. Yet important questions remain to be addressed. A key question is how WNT blockade might affect CD8^+^ T cell function. In our models, pharmacological WNT blockade did not prevent the establishment of CD8^+^ T cell–mediated immune responses. This could be partly explained by the β-catenin independent activity of TCF1 in maintaining the CD8 T cell lineage (Shan et al., 2021). Furthermore, lack of TCF1 does not prevent formation of functional effector and effector memory CD8^+^ T cells; however, at least in the context of viral infection, *Tcf1* null mice lack precursor memory T cells and are susceptible to repeat infection (Jeannet et al., 2010). Whether a similar lack of long-term memory might result from WNT inhibition in cancer, and whether it could be mitigated (for instance by limiting the time of treatment) remains to be investigated. The pancreatic TME is complex, and myeloid cells are key drivers of immune suppression (Du et al., 2021; Zhang et al., 2019b; Zhang et al., 2017). While myeloid cells have no direct expression of WNT components, they may be affected through indirect mechanisms and, over time, acquire a more immunosuppressive phenotype. Further, in this work, we focused on the role of TCF1 in CD4^+^ T cells, as they are the main suppressive lymphocyte populations in pancreatic cancer, based on past work by several groups including ours (Daley et al., 2016; Jang et al., 2017; McAllister et al., 2014; Ochi et al., 2012; Zhang et al., 2014). Yet, CD8^+^ T cells also express TCF1, and these stem-like TILs have been associated with tumor control and response to immunotherapy in other tumor types. However, its function in this cell population in pancreatic cancer remains to be addressed. Lastly, activation of antitumor immunity in pancreatic cancer is challenging due to lack of functional dendritic cells (Hegde et al., 2020); combining WNT inhibition with therapies promoting dendritic cell mobilization, with or without immune checkpoint blockade, might further inhibit tumor growth.

Overall, our work provides new insights into the regulation of the immune suppressive microenvironment in pancreatic cancer and potentially sets the stage for new preclinical studies using combination approaches that include targeting WNT signaling.

## Materials and methods

### Mice

Mice were housed in specific pathogen–free animal facilities of the Rogel Cancer Center at the University of Michigan, overseen by the unit for laboratory animal medicine. *Cd4-CreER*^*T2*^;*Tcf7*^*fl/fl*^ mice were generated by crossing *Tcf7*^*fl/fl*^ (B6(Cg)-Tcf7^tm1Hhx^/J, C57BL/6J strain, stock number 030909; The Jackson Laboratory [Yang et al., 2015]) mice with *Cd4-CreER*^*T2*^ (B6(129X1)-Tg(Cd4-cre/ERT2)11Gnri/J, C57BL/6J strain, stock number 022356; The Jackson Laboratory [Aghajani et al., 2012]) mice. C57BL/6J mice (stock number 000664) were purchased from The Jackson Laboratory.

### Animal experiments

For *Tcf7* depletion in CD4^+^ T cells, *Cd4-CreER*^*T2*^;*Tcf7*^*fl/fl*^ mice and control *Cd4-CreER*^*T2*^;*Tcf7*^*+*^^/^^*+*^ mice (both are C57BL/6J strain) were treated with tamoxifen (4 mg/d for 5 consecutive days) via oral gavage. The mice were then on tamoxifen chow (400 mg/kg) until the end of the experiment. To establish the orthotopic pancreatic cancer model, 5 × 10^4^ 7940b cells (C57BL/6J strain; derived from KPC [*Kras*^*LSL-G12D*^; *Trp53*^*flox/+*^*; Ptf1a-Cre*] tumor [Long et al., 2016]) or 1.25 × 10^5^ mT3-2D cells (C57BL/6J strain; derived from KPC tumor [Boj et al., 2015]) were injected into mice of C57BL/6J strain. Cells were tested for Mycoplasma by the MycoAlert PLUS Mycoplasma Detection Kit (Lonza), and passages 10–15 were used for all experiments. For CD8^+^ T cell depletion, anti-mouse CD8α mAb (BioXCell clone 2.43; 200 μg/mouse) or control IgG (BioXCell clone LTF-2; 200 μg/mouse) was injected i.p. every 3 d. For Ly6G^+^ myeloid-cell depletion, anti-mouse Ly6G mAb (BioXCell clone 1A8; 400 μg/mouse) or control IgG (BioXCell clone 2A3; 400 μg/mouse) was injected i.p. three times a week. For PD-L1 blockade, anti-mouse PD-L1 mAb (BioXcell clone 10F.9G2, 200 μg/mouse) or control IgG (BioXcell clone LTF-2, 200 μg/mouse) was injected i.p. every 3 d. For PORCN inhibition, mice were treated with the PORCN inhibitor (LGK974; Selleckchem, 5 mg/kg) or vehicle (2% DMSO in corn oil) at 12-h intervals via oral gavage. For Th17 depletion, anti-mouse IL-17A mAb (BioXCell clone 17F3; 200 μg/mouse) or control IgG (BioXCell clone MOPC-21; 200 μg/mouse) was injected i.p. every 3 d. For anti-FZD treatment, KC (*Ptf1a-Cre; Kras*^*LSL-G12D*^) mice were treated with anti-FZD antibody Vantictumab (10 mg/kg) or PBS twice per week i.p. for 2 mo before they were sacrificed for study. All animal studies were conducted in compliance with the guidelines of the Institutional Animal Care & Use Committee at the University of Michigan.

### Cell culture

All cell lines were cultured in DMEM (Gibco) with 10% FBS (Gibco) and 1% penicillin streptomycin (Gibco). For IL-17 treatment in BMDM, BM was isolated from the bones of WT C57BL/6J mice and filtered through a 40-μm mesh to obtain single cells. RBC lysis buffer (eBioscience) was used to lyse all the RBCs. BM was cultured in DMEM (Gibco) with 10% FBS (Gibco) and 50% conditioned medium from 7940b cells plus 200 ng/ml recombinant mouse IL-17A (R&D Systems) or PBS. 6 d later, RNA was collected from BMDM. For IL-17 treatment in tumor cells, 7940b cells were treated with 200 ng/ml recombinant mouse IL-17A (R&D Systems) or PBS for 24 h and RNA was collected after treatment. For co-culture experiments, pancreatic tumors from *Cd4-CreER*^*T2*^;*Tcf7*^*fl/fl*^ *or Cd4-CreER*^*T2*^;*Tcf7*^*+*^^/^^*+*^ mice treated with tamoxifen were harvested and disrupted to single cells by mincing the tissue finely using scissors, and further disruption was completed using Collagenase V (Sigma-Aldrich) for 30 min at 37°C while shaking to release the cells. Digestions were subsequently filtered through 500-, 100-, and 40-μm mesh to obtain single cells. CD4^+^ T cells were isolated using CD4 MicroBeads (Miltenyi Biotec). 5 × 10^5^ CD4^+^ T cells were plated in 6-well transwell dishes (0.4 µm pore size; Corning) with 7940b cells plated on the bottom. 24 h after the co-culture, protein and RNA were harvested from 7940b cells. CD4^+^ T cells were stained for surface markers, CD3 and CD4. CD4^+^ T cells were also fixed, permeabilized, and stained for intracellular markers FoxP3 and RORγt. Flow-cytometric analysis was performed on the ZE5 analyzer (Bio-Rad). Data were analyzed using the FlowJo v10.8.0 software.

### scRNA-seq

Human scRNA-seq data were previously published in Steele et al. (2020) (NIH dbGaP database accession #phs002071.v1.p1). Healthy mouse pancreas’ scRNA-seq data were previously published in Kemp et al. (2021) (NCBI Gene Expression Omnibus [GEO] accession no. GSM5011581). To generate the mouse spontaneous PDA scRNA-seq data, pancreatic tumor was harvested from a 6-mo-old KPC mouse. To generate the mouse orthotopic scRNA-seq data, tamoxifen was given to *Cd4-CreER*^*T2*^;*Tcf7*^*fl/fl*^ and *Cd4-CreER*^*T2*^;*Tcf7*^*+*^^/^^*+*^ mice in 5 consecutive days to deplete *Tcf7* in CD4^+^ T cells. The mice were then on tamoxifen chow until the end of the experiment. 5 × 10^4^ 7940b cells were injected into the pancreas of the mice. Pancreatic tumors were harvested and disrupted to single cells by mincing the tissue finely using scissors, and further disruption was completed using Collagenase V (Sigma-Aldrich, 1 mg/ml in RPMI) for 30 min at 37°C while shaking to release the cells. Digestions were subsequently filtered through 500-, 100-, and 40-μm mesh to obtain single cells. Dead cells were removed using the MACS Dead Cell Removal Kit (Miltenyi Biotec). Single-cell cDNA libraries were prepared and sequenced at the University of Michigan Advanced Genomics Core using the 10× Genomics Platform. Samples were run using 50-cycle paired-end reads on the NovaSeq 6000 (Illumina) to a depth of 100,000 reads. The raw data were processed and aligned by the University of Michigan DNA Sequencing Core. Cell Ranger count version 3.1.0 with default settings was used for the spontaneous PDA samples with an initial expected cell count of 10,000. Cell Ranger count version 4.0.0 with default settings was used for orthotopic PDA sample 2038-WD-1 with an initial expected cell count of 20,000. Cell Ranger count version 6.0.1 with default settings was used for orthotopic PDA samples 3421-WD-1 and 3421-WD-2 with an initial expected cell count of 20,000. R version 4.1.0, RStudio version 1.4.1717, R package Seurat version 4.0.1, and R package SeuratObject version 4.0.2 were used for scRNA-seq data analysis (RStudio Team RStudio: Integrated Development for R; http://www.rstudio.com/; R Core Development Team R: A Language and Environment for Statistical Computing; https://www.R-project.org/; Butler et al., 2018). Single-cell data from multiple runs were merged and batch corrected using Seurat’s IntegrateData pipeline (Stuart et al., 2019). The resulting data were filtered to only include cells with at least 100 genes and genes that appeared in more than three cells. Data were normalized using the NormalizeData function with a scale factor of 10,000 and the LogNormalize normalization method. Data were then manually filtered to exclude cells with <900 or >60,000 transcripts and <15% mitochondrial genes. Variable genes were identified using the FindVariableFeatures function. Data were scaled and centered using linear regression of transcript counts. Principal component analysis (PCA) was run with the RunPCA function using the previously defined variable genes. Cell clusters were identified via the FindNeighbors and FindClusters functions using dimensions corresponding to ∼90% variance as defined by PCA. UMAP clustering algorithms were performed with RunUMAP. Clusters were defined by user-defined criteria. The complete R script including figure-specific visualization methods is publicly available on GitHub (https://github.com/PascaDiMagliano-Lab/). Raw data for mouse spontaneous PDA (KPC) is available on NCBI GEO (GSM6127792). Raw data for mouse orthotopic PDA is available on NCBI GEO (GSE199436).

### IHC and IF

Pancreatic tissues were fixed in 10% neutral-buffered formalin (FisherBrand) and then embedded in paraffin and sectioned into slides. For IHC, fresh-cut paraffin slides were rehydrated using two series of xylene, two series of 100% ethanol, and then two series of 95% ethanol. Water was used to wash all residues from previous washes. Antigen retrieval was performed using Antigen Retrieval CITRA Plus (BioGenex) and microwaved for a total of 8 min. Upon cool-down, tissue was blocked using 1% BSA in PBS for 30 min, and then primary antibodies in Table S1 were used at their corresponding dilutions. Biotinylated secondary antibodies were used in 1:300 dilution. Following the secondary antibody incubation, the tissue was incubated for 30 min with the ABC reagent from VECTASTAIN Elite ABC kit, peroxidase (Vector Laboratories). Then it was developed using DAB (Vector). For IF, Alexa fluor secondary antibodies (1:300, Invitrogen) were used. Prolong Diamond Antifade Mountant with DAPI (Invitrogen) was used for nuclei staining. The Tyramide SuperBoost Kit (Invitrogen) was used in IF when primary antibodies raised in the same species were used. Images were taken with an Olympus BX-53 microscope, Olympus DP80 digital camera, and Olympus cellSens standard software. Some IF images were acquired using the Leica Stellaris 5 confocal microscope (Leica Microsystems).

### In situ hybridization (ISH) with co-IF

ISHs were performed with the RNA Scope Multiplex Fluorescent Detection Kit (Advanced Cell Diagnostics) according to the manufacturer’s protocol. A probe for *Tcf7* (557491; Advanced Cell Diagnostics) was used. Briefly, freshly cut paraffin-embedded sections were baked for 1 h at 60°C prior to staining. Slides were then deparaffinized and treated with hydrogen peroxide for 10 min at room temperature. Target retrieval was performed in a water steamer boiling for 15 min and then slides were treated with the ProteasePlus Reagent (Advanced Cell Diagnostics) for 30 min. Following this, the RNA scope probe was hybridized for 2 h at 40°C. The signal was amplified using the AMP materials provided in the ACD Multiplex Kit (Advanced Cell Diagnostics). The signal was then developed with an HRP channel. Once completed, the samples were washed in PBS and then blocked for 1 h with 5% donkey serum at room temperature. Primary antibodies were incubated overnight at 4°C. Secondary antibodies (1:300 in blocking buffer) were incubated for 1 h at room temperature and samples were washed three times in PBS. Slides were counterstained with DAPI and mounted with ProLong Gold Antifade Mountant (Thermo Fisher Scientific). Images were acquired using the Leica Stellaris 5 confocal microscope (Leica Microsystems).

### Flow cytometry and FACS

Pancreas was harvested and disrupted to single cells as described above. Cells were stained for surface markers using antibodies listed in Table S1. Cells were also fixed and permeabilized before intracellular staining using antibodies listed in Table S1. Flow-cytometric analysis was performed on the ZE5 analyzer (Bio-Rad). Data were analyzed using the FlowJo v10.8.0 software. FACS was performed using the Propel Bigfoot Spectral Cell Sorter (Thermo Fisher Scientific).

### Magnetic cell separation

For CD4^+^ T cell isolation from spleens and lymph nodes, samples were dissociated by forcing through a 40-µm cell strainer using a plunger from a sterile 3 cc syringe in a circular motion. Single-cell suspension and blood samples were incubated with ACK (Ammonium-Chloride-Potassium) Lysing Buffer to remove RBCs and then washed before CD4^+^ T cell isolation using CD4^+^ T Cell Isolation Kit, mouse (Miltenyi Biotec). For CD8^+^ T cell isolation from spleens, the same protocol with CD8a^+^ T Cell Isolation Kit, mouse (Miltenyi Biotec) was used; for CD8^+^ T cell isolation from tumors, single-cell suspensions were obtained using Collagenase V protocol described above and CD8 (TIL) MicroBeads, mouse (Miltenyi Biotec) was used to purify tumor-infiltrating CD8^+^ T cells.

### CyTOF

Pancreas was harvested and disrupted to single cells as described above. Cells were washed twice in PBS, and Cell-ID cisplatin (1.67 μmol/liter) was used for 5 min at room temperature as a viability marker. Surface and intracellular staining (Table S1) was performed as detailed in manufacturer instructions (Fluidigm). Cells were shipped in intercalator buffer on ice overnight to Indiana University Simon Cancer Center Flow Cytometry Core where the sample preparation was finalized and CyTOF2 Mass Cytometer analysis was performed. Data analysis was performed using the Premium CytoBank Software (cytobank.org). Live singlets were gated using the DNA Intercalator Ir191, event length, and Cisplatin Pt195.

### Western blot

Cells were lysed using radioimmunoprecipitation assay buffer (Sigma-Aldrich) with protease and phosphatase inhibitors (Sigma-Aldrich). Protein was quantified and the same amount of protein was loaded to the wells in a 4–15% SDS-PAGE gel (BioRad). Protein was transferred to a polyvinylidene fluoride membrane (BioRad) that was blocked with 5% milk for 1 h at room temperature and then incubated with primary antibodies listed in Table S1 overnight. HRP-conjugated secondary antibodies (1:5,000) were used and detected by using the SuperSignal West Femto Maximum Sensitivity Substrate (Thermo Fisher Scientific). The bands were visualized using the ChemiDoc Imaging System (BioRad).

### qRT-PCR

RNA was extracted using the RNeasy Mini Kit (Qiagen). RNA samples went through RT-PCR using the High-Capacity cDNA Reverse Transcription Kit (Applied Biosystems). cDNA samples for quantitative Real-Time PCR were prepared using a mix of 1× Fast-SYBR Green PCR Master Mix (Applied Biosystems) and the primers listed in Table S2. *Ppia* was used as the housekeeping control.

### Statistical analyses

GraphPad Prism 9 software was used for all statistical analyses. All data were presented as means ± SD. Two-tailed Student *t*-test (two groups) and one-way ANOVA with Tukey test (three and four groups) were performed for comparison between groups. P < 0.05 was considered significant.

### Online supplemental material

Primary antibodies used in IHC/IF, flow cytometry, CyTOF, and Western blot are included in Table S1. Primer sequences used for qRT-PCR are listed in Table S2. Fig. S1 shows scRNA-seq analysis of mouse and human pancreas and pancreatic cancer, as well as the effect of Tcf7 inactivation on expression of WNT target genes in spleen T cells. Fig. S2 contains data on tumor characteristics and WNT signaling components. Fig. S3 includes scRNA-seq data of mouse orthotopic pancreatic tumors. Fig. S4 shows that depletion of combination of *Tcf7* inactivation in T cells and depletion of MDSCs fails to improve tumor inhibition. Fig. S5 shows CD8^+^ T cell infiltration upon pharmacological inhibition of WNT signaling.

## Supplementary Material

Table S1 includes primary antibodies used in IHC/IF, flow cytometry, CyTOF, and Western blot.

Table S2 lists primer sequences used for qRT-PCR.

SourceData F5 contains original blots for Fig. 5.
