# Supplementary material for: WNT signaling in the tumor microenvironment promotes immunosuppression in murine pancreatic cancer
Source: J Exp Med. 2022 Oct 14;220(1):e20220503. doi: 10.1084/jem.20220503 (PMC9577101; doi:10.1084/jem.20220503)
Supplement: Table S1 — includes primary antibodies used in IHC/IF, flow cytometry, CyTOF, and Western blot. [file JEM_20220503_TableS1.docx]

Supplementary Table 1

| Antibody | Supplier | Catalog number/Clone | Dilution | Label |
| --- | --- | --- | --- | --- |
| **IHC** |  |  |  |  |
| CD4 | Abcam | ab183685 | 1:100 |  |
| CD8 | Cell Signaling | 98941 | 1:400 |  |
| Cleaved Caspase 3 | Cell Signaling | 9661 | 1:400 |  |
| FoxP3 | Cell Signaling | 12653 | 1:100 |  |
| RORγt | BD Pharmingen | 562607 | 1:200 |  |
| Ki67 | Abcam | ab15580 | 1:100 |  |
| PDGFRβ | Abcam | ab32570 | 1:200 |  |
| Podoplanin | BioLegend | 127403 | 1:1000 |  |
| **IF** |  |  |  |  |
| Human CD4 | Cell Signaling | 48274 | 1:50 |  |
| Human CD8 | Cell Signaling | 70306 | 1:200 |  |
| Human TCF1 | R&D Systems | AF5596 | 1:50 |  |
| αSMA | Sigma-Aldrich | A2547 | 1:1000 |  |
| Arginase 1 | Cell Signaling | 93668 | 1:100 |  |
| CD4 | Abcam | ab183685 | 1:100 |  |
| CD8 | Cell Signaling | 98941 | 1:400 |  |
| CK19 | Iowa Developmental Hybridoma Bank |  | 1:50 |  |
| Cleaved Caspase 3 | Cell Signaling | 9661 | 1:400 |  |
| E-Cadherin | Cell Signaling | 14472 | 1:50 |  |
| F4/80 | Cell Signaling | 70076 | 1:200 |  |
| Granzyme B | Cell Signaling | 17215 | 1:100 |  |
| **Flow cytometry/FACS** |  |  |  |  |
| CD3 | BioLegend | 17A2 | 1:100 | APC |
| CD4 | BD Pharmingen | RM4-5 | 1:100 | Pacific Blue |
| CD8 | BD Pharmingen | 53-6.7 | 1:100 | APC-Cy7 |
| FoxP3 | Invitrogen | FJK-16s | 1:100 | AF488 |
| RORγt | BD Pharmingen | Q31-378 | 1:100 | PE |
| CD45 | BD Horizon | 30-F11 | 1:100 | BV510 |
| CD11b | BD Pharmingen | M1/70 | 1:100 | APC-Cy7 |
| F4/80 | Invitrogen | BMB | 1:100 | PE-Cy5 |
| Ly6G | BD Pharmingen | 1A8 | 1:100 | FITC |
| Ly6C | BD Pharmingen | AL-21 | 1:100 | PE |
| PD-L1 | BD Pharmingen | MIH5 | 1:100 | PE |
| EpCAM | Invitrogen | G8.8 | 1:100 | APC |
| **CyTOF** |  |  |  |  |
| EpCAM | Fluidigm | G8.8 | 1:100 | 165Ho |
| CD45 | Fluidigm | 30-F11 | 1:200 | 089Y |
| CD19 | Fluidigm | 6D5 | 1:200 | 149Sm |
| NK1.1 | Fluidigm | PK136 | 1:100 | 170Er |
| TCRγδ | Fluidigm | GL3 | 1:100 | 159Tb |
| CD3e | Fluidigm | 145-2C11 | 1:100 | 152Sm |
| CD4 | Fluidigm | RM4-5 | 1:100 | 145Nd |
| CD8a | Fluidigm | 53-6.7 | 1:100 | 168Er |
| CD25 | Fluidigm | 3C7 | 1:100 | 151Eu |
| FoxP3 | Fluidigm | FJK-16s | 1:100 | 158Gd |
| TIM3 | Fluidigm | RMT3-23 | 1:100 | 162Dy |
| CD11b | Fluidigm | M1/70 | 1:300 | 143Nd |
| CD11c | Fluidigm | N418 | 1:100 | 209Bi |
| Ly6G | Fluidigm | 1A8 | 1:400 | 141Pr |
| Ly6C | Fluidigm | HK1.4 | 1:500 | 150Nd |
| F4/80 | Fluidigm | BM8 | 1:100 | 146Nd |
| iNOS | Fluidigm | CXNFT | 1:100 | 161Dy |
| Arginase 1 | Fluidigm | 14D2C43 | 1:100 | 164Dy |
| CD206 | Fluidigm | C068C2 | 1:200 | 169Tm |
| CD274 | Fluidigm | 10F.9G2 | 1:100 | 153Eu |
| **WB** |  |  |  |  |
| PD-L1 | BioXCell | 10F.9G2 | 8 µg/mL |  |
| Vinculin | Cell Signaling | 4650 | 1:1000 |  |
