# Supplementary material for: WNT signaling in the tumor microenvironment promotes immunosuppression in murine pancreatic cancer
Source: J Exp Med. 2022 Oct 14;220(1):e20220503. doi: 10.1084/jem.20220503 (PMC9577101; doi:10.1084/jem.20220503)
Supplement: Table S2 — lists primer sequences used for qRT-PCR. [file JEM_20220503_TableS2.docx]

Supplementary Table 2

|  | Forward | Reverse |
| --- | --- | --- |
| *Axin2* | GCCAATGGCCAAGTGTCTCT | GCGTCATCTCCTTGGGCA |
| *Cd274* | GCTGAAGTCAATGCCCCATA | TCCACGGAAATTCTCTGGTTG |
| *Foxp3* | CACCTATGCCACCCTTATCCG | CATGCGAGTAAACCAATGGTAGA |
| *Il4* | CCCCAGCTAGTTGTCATCCTG | CGCATCCGTGGATATGGCTC |
| *Il10* | GCTATGCTGCCTGCTCTTACT | CCTGCTGATCCTCATGCCA |
| *Il17a* | AAGGCAGCAGCGATCATCC | GGAACGGTTGAGGTAGTCTGAG |
| *Ifng* | TCAAGTGGCATAGATGTGGAAGAA | TGGCTCTGCAGGATTTTCAGT |
| *Ppia* | TCACAGAATTATTCCAGGATTCATG | TGCCGCCAGTGCCATT |
| *Tcf7* | AACTGGCCCGCAAGGAAAG | CTCCGGGTAAGTACCGAATGC |
| *Tgfb* | TGACGTCACTGGAGTTGTACGG | GGTTCATGTCATGGATGGTGC |
| *Lef1* | TGTTTATCCCATCACGGGTGG | CATGGAAGTGTCGCCTGACAG |
