# Supplementary figures and images for: WNT signaling in the tumor microenvironment promotes immunosuppression in murine pancreatic cancer
Source: J Exp Med. 2022 Oct 14;220(1):e20220503. doi: 10.1084/jem.20220503 (PMC9577101; doi:10.1084/jem.20220503)

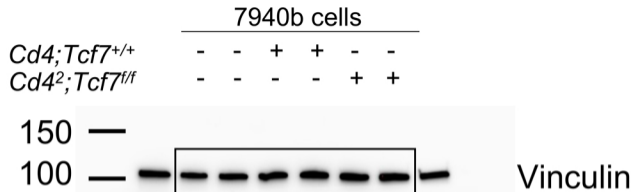

Lower exposure

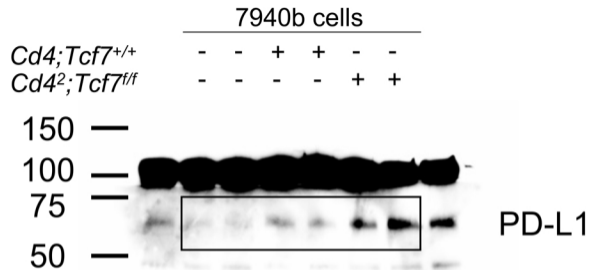

Higher exposure

Supplement: SourceData F5 — contains original blots for Fig. 5. [file JEM_20220503_SourceDataF5.pdf]
